# The black honey bee genome: insights on specific structural elements and a first step towards pangenomes

**DOI:** 10.1186/s12711-024-00917-3

**Published:** 2024-06-28

**Authors:** Sonia E. Eynard, Christophe Klopp, Kamila Canale-Tabet, William Marande, Céline Vandecasteele, Céline Roques, Cécile Donnadieu, Quentin Boone, Bertrand Servin, Alain Vignal

**Affiliations:** 1 GenPhySE, Université de Toulouse, INRAE, INPT, INP-ENVT, Castanet Tolosan, France; 2 Sigenae, MIAT, INRAE, Castanet Tolosan, France; 3 CNRGV, INRAE, Castanet Tolosan, France; 4 INRAE, US 1426, GeT-PlaGe, Genotoul, Castanet-Tolosan, France

## Abstract

**Background:**

The honey bee reference genome, HAv3.1, was produced from a commercial line sample that was thought to have a largely dominant *Apis mellifera ligustica* genetic background. *Apis mellifera mellifera*, often referred to as the black bee, has a separate evolutionary history and is the original type in western and northern Europe. Growing interest in this subspecies for conservation and non-professional apicultural practices, together with the necessity of deciphering genome backgrounds in hybrids, triggered the necessity for a specific genome assembly. Moreover, having several high-quality genomes is becoming key for taking structural variations into account in pangenome analyses.

**Results:**

Pacific Bioscience technology long reads were produced from a single haploid black bee drone. Scaffolding contigs into chromosomes was done using a high-density genetic map. This allowed for re-estimation of the recombination rate, which was over-estimated in some previous studies due to mis-assemblies, which resulted in spurious inversions in the older reference genomes. The sequence continuity obtained was very high and the only limit towards continuous chromosome-wide sequences seemed to be due to tandem repeat arrays that were usually longer than 10 kb and that belonged to two main families, the 371 and 91 bp repeats, causing problems in the assembly process due to high internal sequence similarity. Our assembly was used together with the reference genome to genotype two structural variants by a pangenome graph approach with Graphtyper2. Genotypes obtained were either correct or missing, when compared to an approach based on sequencing depth analysis, and genotyping rates were 89 and 76% for the two variants.

**Conclusions:**

Our new assembly for the *Apis mellifera mellifera* honey bee subspecies demonstrates the utility of multiple high-quality genomes for the genotyping of structural variants, with a test case on two insertions and deletions. It will therefore be an invaluable resource for future studies, for instance by including structural variants in GWAS. Having used a single haploid drone for sequencing allowed a refined analysis of very large tandem repeat arrays, raising the question of their function in the genome. High quality genome assemblies for multiple subspecies such as presented here, are crucial for emerging projects using pangenomes.

**Supplementary Information:**

The online version contains supplementary material available at 10.1186/s12711-024-00917-3.

## Background

The honey bee *Apis mellifera* was originally found in Europe, Africa, and the Middle East, with the most eastern limit of its natural distribution situated in western Afghanistan, until a new subspecies was discovered in Kazakhstan [1]. The evolutionary origin of *Apis mellifera* is still unclear, with a possible origin in Eastern Africa or the Middle East, followed by colonization of Europe through different routes, leading to high genetic differentiation between geographically close populations or subspecies, namely *A. m. mellifera* (otherwise referred to as M-type) in western Europe on one side and *A. m. ligustica* from Italy or *A. m. carnica* (known as C-type) from eastern Europe on the other [2–5]. However, although *A. m. mellifera* is the original subspecies found in western Europe, it has become commonplace amongst breeders, in order to increase production or to facilitate the handling of colonies, to import other subspecies, mainly *A. m. ligustica* from Italy, *A. m. carnica* from Slovenia, and *A. m. caucasica* from Georgia. These are either to be bred as pure lines or as hybrids generated by artificial or directed insemination [6, 7]. As a consequence, these imported subspecies and hybrid lines mate naturally to local *A. m. mellifera* populations, threatening them and prompting the establishment of conservation programmes [8]. However, although it has been replaced in the majority of large professional beekeeper’s facilities by imported honey bees, *A. m. mellifera* is still used by specialised breeders.

The honey bee reference genome, whose first version was obtained in 2006 [9], was updated twice: first in 2014 [10] and a second time in 2019, using long-read sequencing together with Hi-C chromatin interaction and BioNano Optical maps for a chromosome-scale assembly [11]. The sample used for this reference genome was from a commercial line (DH4) that was not precisely genetically defined, but is thought to be mainly of *A. m. ligustica* descent [9]. As a consequence, the genome of the genetically distinct *A. m. mellifera* may not be accurately represented and future pangenome approaches, which have been shown in other species to expand the number of genomic regions available for analysis [12, 13], would benefit from a high-quality assembly for this important subspecies.

To ensure a faithful representation of the *A. m. mellifera* subspecies genetic background, an individual from the black bee conservatory “Association Conservatoire de l’Abeille Noire Bretonne” in the island of Ouessant, France, was selected for sequencing. Ouessant is a very small island (15.5 km^2^), located 20 km off the coast of Brittany, in which a conservation population was set up starting in 1987. Any further imports of other honey bees are banned since 1991. Mitochondrial DNA analyses have shown a low haplotype diversity and the presence of only the M-type in this population [14]. As expected from such a small population, microsatellite analysis has shown low diversity [15].

Until the latest update [11], the current honey bee genome sequence, Amel4.5 [10], suffered from imperfections, having numerous gaps in the assembly and possible sequence inversions. In order to construct a new *A. m. mellifera* genome assembly with improved continuity, we used the Pacific Biosciences long-read technology and produced all sequence reads from a single haploid drone to avoid assembly problems due to polymorphism. To order and orient our contigs along the chromosomes, we used published sequencing reads from drones originating from three colonies that had previously been used to map meiotic crossovers and non-crossovers in the honey bee [16], allowing also for the production of an updated genetic map and a re-estimation of the honey bee recombination rate.

Our analyses of the assembly allowed the detection of a major family of tandem repeats, running in some instances over more than 10 kb, and found at the ends of most sequence contigs. Our assembly allows for the first-time to perform detailed analyses of structural rearrangements, including at the population level, between the genomes of *A. m. ligustica* and other C-type honey bees that are used by the majority of beekeepers, and that of the M-type subspecies *A. m. mellifera* black bee.

## Methods

### Sampling, DNA extraction, and PacBio long-read sequencing

Candidate drones for sequencing were sampled at the larval or pupae stage from the black bee conservatory on the island of Ouessant, Brittany, France, and extractions were performed from several samples in order to select the best DNA quality in terms of molecular weight and quantity. Each sample was ground using a potter (see Additional file 1 Figure S1) and DNA extraction was performed using the QIAGEN Genomic-tips 100/G kit (Cat No./ID: 10243), following the tissue protocol extraction (see supplementary methods). DNA for sequencing was obtained from a single drone OUE7B (see Additional file 1 Figure S2). Library preparation and sequencing were performed at the GeT-PlaGe core facility, INRAE Toulouse, following the manufacturer’s instructions for “Shared protocol-20 kb Template Preparation Using BluePippin Size Selection system (15 kb size Cutoff)”. At each step, DNA was quantified using the Qubit dsDNA HS Assay Kit (Life Technologies). DNA purity was tested using a nanodrop (Thermofisher) and size distribution and degradation was assessed using the Fragment analyzer (AATI) High Sensitivity Large Fragment 50 kb Analysis Kit. Purification steps were performed using 0.45X AMPure PB beads (PacBio). Thirty µg of DNA was purified to perform three libraries. Using SMRTBell template Prep Kit 1.0 (PacBio), a DNA and end damage repair step was performed on 15 µg of unshared sample. Then blunt hairpin adapters were ligated to the libraries. The libraries were treated with an exonuclease cocktail to digest unligated DNA fragments. A size selection step using a 7 kb (library 1) or 9 kb (libraries 2 and 3) cutoff was performed on the BluePippin Size Selection system (Sage Science) with 0.75% agarose cassettes, Marker S1 high Pass 15-20 kb. Conditioned Sequencing Primer V2 was annealed to the size-selected SMRTbells. The annealed libraries were then bound to the P6-C4 polymerase using a ratio of polymerase to SMRTbell at 10:1. Then, after a magnetic bead-loading step (OCPW), SMRTbell libraries were sequenced on 36 SMRTcells on a RSII instrument from 0.05 to 0.2 nM, with a 360 min movie.

### Assembly into contigs and alignment to Amel4.5 for chromosome assignments

Raw reads were assembled with Canu 1.3 [17] using standard parameters and a first polishing of the assembly was done with quiver (version SMRT_Link v4.0.0) using standard parameters. The contigs obtained after the assembly step were aligned to the Amel4.5 reference genome using LAST v956 [18].

### Alignment of Illumina sequencing reads and SNP calling for crossing over analysis

All the Illumina paired-end sequences from Liu et al. [16] were downloaded from the NCBI SRA project SRP043350 (see Additional file 2 Table S1). The reads were aligned to the assembled contigs with BWA MEM v0.7.15 [19], duplicate reads were removed with Picard (v2.1.1; http://picard.sourceforge.net), and local realignment and base quality score recalibration (BQSR) was performed using GATKv3.7 [19]. SNPs were called in each drone independently with GATK HaplotypeCaller and consolidated into a single set of master sites, from which all individuals were genotyped with GATK GenotypeGVCFs (see scripts in supplementary material). Hard filtering of variants was performed with the following filters: FS > 60, MQ < 50 and SOR > 3; any SNP with missing genotypes were filtered out. Further quality controls were applied and, for each colony, SNPs falling into any of the following categories were discarded: (i) non-polymorphic SNPs in the colony, (ii) homozygous SNPs in the queen, (iii) heterozygous SNPs in drones, (iv) SNPs that appeared inconsistent with the observations in the two other colonies and (v) SNPs showing inconsistent allelic versions between queen and drone genotypes.

### Phasing and detection of recombination events

For each colony and informative SNP, genotyping results were used to define genotype vectors across all drones for the colony. Identical genotype vectors that followed one another within the same contig defined a segment with no observed crossing over in the drones of the colony and were grouped into bins. Not having access to grand-parental genotypes, genotype phase between two successive bins within a contig was determined by finding which out of the two possible inverse vectors minimised the number of recombination events. Non-crossing-over gene conversion events, which can be misinterpreted as double recombination events and that occur usually on short DNA fragments, often shorter than a few kb [16], were removed to avoid inflating the size of the genetic map. Non-crossing-over gene conversion events were identified as: (i) bins of length shorter than 2 kb and occurring between two identical bins, or (ii) bins of length shorter than 2 kb for which the number of recombination events happening within this bin was higher than the number of recombination events needed to go from the bin before to the bin after it. Bins detected as non-crossing-over gene conversions were merged with their two identical surrounding bins. Both phasing and putative non-crossing over identification were performed iteratively from one bin to the next and independently for each colony. As a consequence, a set of phased vectors that minimised recombination events was obtained for each contig for each colony.

### Scaffolding contigs into chromosomes

Using the a priori assignment of contigs to chromosomes by alignment to Amel4.5 as a starting point, contigs were ordered and oriented iteratively in order to minimise the number of recombination events between the genotype vectors defined at their extremities. Contig scaffolding was first performed using the data for each colony separately and was thereafter confirmed using markers informative across all three colonies.

### Correction of the assembly with Illumina reads

Genomic DNA from the same individual as used for the PacBio sequencing was sequenced with an Illumina NovaSeq6000 instrument, producing over 28,000,000 reads (estimated raw sequencing depth = 37 X), NCBI SRA accession SRR15173860. These were aligned on the assembled genome with BWA MEM version 0.7.12-r1039 [20] using standard parameters. Variant detection was done with freebayes version 1.1.0 [21] and filtered to retain only those with a minimum quality score of 20 and '1/1' genotype or '0/1' with no read supporting the reference allele. Finally, corrections to the genome assembly were done when alternative alleles were found in the VCF file using vcf-consensus from the vcftools package (version 0.1.12a) [22] with standard parameters.

### Comparison with Amel 4.5 and HAv3.1 assemblies

Estimation of recombination rate and positioning recombination events along the Amel4.5 and AMelMel1.1 assemblies was done using the same procedure as used for the de-novo assembly. GC content and sequence coverage for the queens’ genotypes in AMelMel1.1 were measured in 0.5 Mb windows and the recombination rates were estimated using a script from Petit et al. [23] over 1 Mb windows. Completeness of the assemblies was estimated with BUSCO 3.0.2 [24] using OrthoDB v9.1 single-copy orthologs [25], from the Metazoa (n = 978) and Hymenoptera (n = 4415) BUSCO core set. Alignments of AMelMel1.1 to Amel4.5 and to HAv3.1 were done using LAST v956 [18]. Standard output psl files were produced to keep all alignments related to repeat elements, together with psl files from split alignments [18], corresponding to one-to-one alignments. Dotplot visualisation of alignments were produced with custom scripts, available at https://github.com/avignal5/PacificBee/tree/main. Inversions between the two genome assemblies were detected in the split alignment psl file. Liftovers of the HAv3.1 gtf and gff annotation to produce files with AMelMel1.1 annotation coordinates were done using CrossMap [26] and the chained alignment format output from the AMelMel1.1 to HAv3.1 LAST alignments.

### Analysis of repeat elements

Analysis of tandem repeats was done with Tandem Repeat Finder v4.09 (TRF) [27], setting the maximum period size to 2000 bp. The two major classes of repeat sizes, the 91 bp repeat and the 371 bp repeat, were analysed by aligning all repeats within a class size with MAFFT v7.313 [28]. Sequences reported by TRF from different parts of the genome start at different positions of the repeated element that was detected and to address this, the multifasta alignments produced by MAFFT were processed with a custom script to determine an identical arbitrary start point for all sequences before performing a second alignment with MAFFT. Phylogenetic trees were constructed using Jalview v2.11.2 [29] with the average distance option. Consensus sequences from all sequences selected within the groups defined based on the phylogenetic trees were used for a BLAST search in the AMelMel1.1 assembly and hits following one another at distances shorter than the repeat period size were grouped together. Finally, the previously described monomer consensus sequences, accession X57427.1 for *Alu*I and X89530.1 for *Ava*I, were used to detect their presence in the assembly by BLAST.

### Analysis of indels in populations

Indels were detected by aligning the two genomes HAv3.1 and AMelMAl1.1 to one another with minimap2 [30], followed by variant calling with SVIM-asm [31]. Two nuclear mitochondrial DNA (NUMT) were then selected for genotyping in a set of 80 haploid males, representing the three major European bee subspecies: *A. m. mellifera* (n = 35), *A. m. ligustica* (n = 30), and *A. m. caucasica* (n = 15) (see Additional file 2 Table S2). All 80 samples were aligned to both assemblies, as described in Wragg et al. [6], and sequencing depth was estimated using SAMtools [32]. Individual genotypes in the sequencing data were determined for two selected indels by two methods. One method consisted of using GraphTyper2 [33] to detect breakpoints due to insertions, deletions, or inversions in the pangenome graph built with SVIM-asm using the two assemblies, HAv3.1 and AMelMel1.1. The other method consisted in using sequencing depths as an indication of presence or absence of indels. For a given indel and for each sample, the sequencing depth for the alignments on the genome in which the indel is present was calculated and compared to the sequencing depth of the sequences that flanked the indel on both sides. Normalisation was done by calculating the ratio between sequencing depth in the indel and in the flanking sequences. Determination of presence or absence of the indels was then done by K-means clustering with K = 2. The vcf file produced by SVIM-asm was used to estimate the number and size distribution of indels that were larger than 40 bp. Bedtools v2.30.0 was used to check that indels did not overlap with contig boundaries in one or the other assembly and to search for overlaps between indels and exons in the GCF_003254395.2_Amel_HAv3.1_genomic.gff annotation file that was downloaded from NCBI.

## Results

### PacBio long-read sequencing and assembly into contigs

All long-read sequence data came from a single haploid drone that was selected amongst several tested based on having the highest DNA concentration and a peak of DNA fragment length at 35 kb (see Additional file 1 Figure S2). A high proportion of reads exceeded 10 kb and a few reads were longer than 70 kb. Their size distribution is shown in Additional file 1 Figure S3 and S4. After assembly, a total of 200 contigs (gap-free sequence tracts) was obtained. The longest contig was 11.6 Mb and the N50 contig size was 5.1 Mb (see Additional file 2 Table S3 and see Additional file 1 Figure S5). These results are a major improvement in comparison to the 46 kb N50 contig of Amel4.5 and quite similar to the N50 contig of 5.4 Mb observed in the HAv3.1 assembly [11]. Analysis with BUSCO showed that overall, AMelMel1.1 had a slightly larger gene content than both Amel 4.5 and the most recently published reference assembly AmelHAv3.1 [11] (see Additional file 2 Table S4).

### Chromosomal assignment and contig ordering with crossing-over data

A priori chromosomal assignment of contigs was done by alignment to the Amel4.5 assembly using LAST v956 [18]. Out of the 200 contigs, 110 aligned successfully. Crossing-over data to confirm chromosome assignment and the order of contigs along chromosomes was obtained by using the reads from the sequencing of 43 drones from the three colonies that were initially used to estimate recombination rate in honey bee [16]. Briefly, this data set contains sequence data for three queens and their drone offspring (15 to 13 depending on the colony). Three of the drones of colony 1 were sequenced in duplicate and were used for quality control of SNP calling. Aligning these reads to our contigs allowed the detection of 2,103,924 SNPs, on 176 contigs before quality control. Out of these, approximately 64.5% were discarded due to lack of polymorphism across the three colonies, 1% for being homozygous in the queens, and 1% for being heterozygous in the drones. Furthermore, 0.2% of the SNPs were discarded for being inconsistent between the three drone replicates and 0.4% were discarded for having allelic inconsistencies between queen and drones of the same colony. After all the quality controls and for each of the three colonies, 687,699, 698,123, and 672,728 reliable SNPs (approximately 32% of the initial SNPs), were detected for the three colonies, on 114, 112, and 113 contigs respectively (see Additional file 1 Figure S6). In total 120 contigs were at least partially informative across the colonies, with 104 contigs informative in the three colonies and 16 for only one or two. A total of 114,754 polymorphic SNPs was present in the 104 contigs that were informative across all three colonies (see Additional file 1 Figure S6). Genotype vectors for each SNP across colony drones were then defined, allowing for detection of within-contig cross-overs (see Additional file 1 Figure S7). Genotype vectors from the ends of contigs were then used to join contig ends together by finding for each contig end, the best corresponding end from another contig that had either the same genotype vector or a genotype vector that presented a minimal number of cross-overs (see Additional file 1 Figure S7). To minimize the number of comparisons, the a priori chromosomal assignment by alignment to Amel4.5 (see above) was used.

One hundred and two contigs out of the 110 with chromosome assignment by sequence similarity to Amel4.5 had SNP genotype data and were thus informative for cross-over detection. At least one cross-over event, as evidenced by the presence of at least 2 genotype vector bins, could be detected within 86 of these contigs, thus allowing for their orientation. The remaining 16 contigs were oriented based on their alignment to Amel4.5. All these contigs were small, except for one contig on chromosome 7. For this contig, despite its large size, close to 2.4 Mb, it was difficult to orientate using the genetic map, as no cross-over could be detected due to an unusually low number of SNPs and a very low local recombination rate. Moreover, its orientation could not be deduced from Amel4.5 or even from the more recent assembly HAv3.1, as both possible orientations induced large inversions when compared to these other two assemblies. Contigs assigned to chromosomes by alignment only (8 contigs) or by crossing-over data alone (16 contigs), were assigned to their chromosomes, but at an unknown (unlocalised) position. All remaining 72 contigs were considered unplaced (see Additional file 1 Figure S6).

### Tandem repeats at contig boundaries and orientation of a large inversion on chromosome 7

**Fig. 1:**
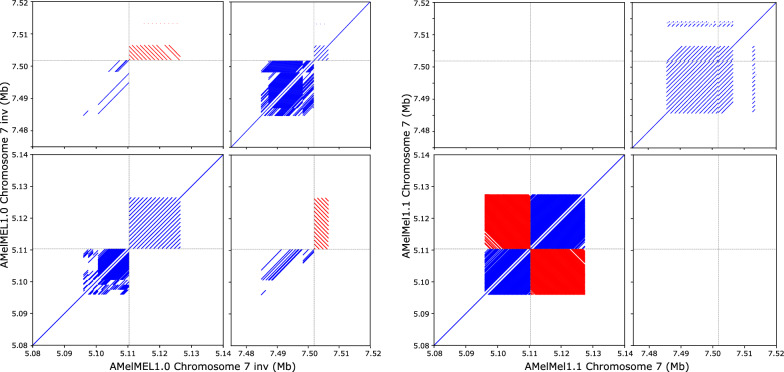
Orientation of the AMelMel1.1 contig, presenting an inversion on chromosome 7 when compared to HAv3.1. The repeats present at the boundary between the contigs were used to orient the AMelMel1.1 contig on chromosome 7. Assemblies with one or the other orientation of the contig were self-aligned with LAST. Left: orientation from AMelMel1.0. Right: orientation from AMelMel1.1. For each pair of alignments, only the junction between contigs are shown: the two ends of the contig to orient, the end of the previous, and the start of next contigs. Results clearly show that the orientation in AMelMel1.1 is the correct one

With long-read data, sequence contigs are large but they still don’t cover the entire length of chromosomes, with the exception of chromosome 16. When analysing the contig ends, we found that almost all were composed of tandem repeats arrays that were usually longer than the read lengths, thus preventing assembly. To orientate the large contig on chromosome 7, positioned as 5th in order along the chromosome by the cross-over data, we took advantage of the fact that the repeat elements detected by TRF and that were present at both extremities of the contig had different period sizes (258 and 1296 bp) and consensus sequences. These were compared to the proximal repeats of the 4th and the 6th contigs of chromosome 7. Interestingly, a tandem repeat element of 258 bp was detected at the end of the 4th contig, and another one of 1296 bp at the end of the 6th contig. Both had period sizes that were identical to the extremities of the 5th contig, suggesting the correct orientation of the 5th contig. Correspondence between these contig ends was further examined by pairwise alignment of the repeat sequences with NCBI BLAST. Identity was 100% between the sequences of identical period sizes, whereas no significant similarity could be found between the others (Fig. 1 and see Additional file 2 Table S5), thus confirming the orientation of the contig. Dotplots that compare AMelMel1.1 and HAv3.1 are shown in Additional file 3 Figure S22 and suggest a very small number of discrepancies, the major one residing on chromosome 7.

### Telomere and centromere consensus sequences

**Fig. 2:**
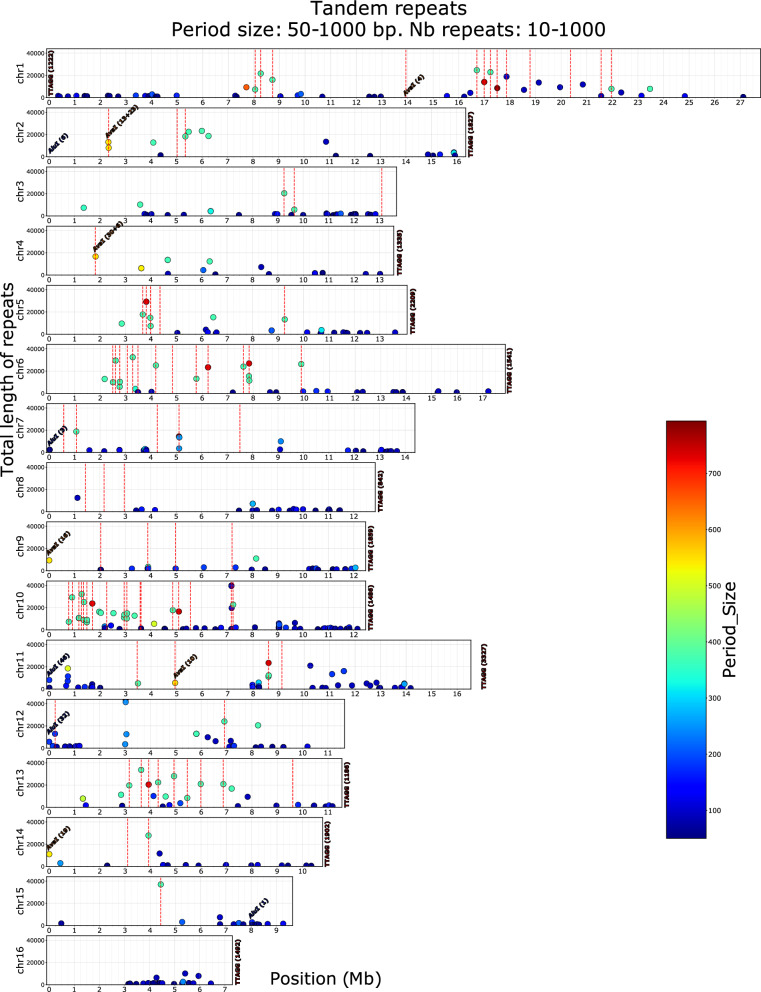
Tandem repeats of period size 90–371 bp detected in the AMelMel1.1 assembly. The colour scale represents the period size of the repeat elements and the Y axis the total length of the repeat array. Vertical dotted lines represent the contig boundaries in the AMelMel1.1 assembly. The position of *Alu*I and *Ava*I repeats are indicated with the number of repeats in parentheses. The figure shows clearly that most contigs are separated by tandem repeats of period size close to 371 bp, of length in the order of 10 kb or more. See also Additional file 1: Fig S11 for repeats of longer period size (1000–2000 bp). Although not represented on the graph (period size = 5), TTAGG telomere repeats are indicated with the number of repeats in parentheses, when present at a chromosome end

The presence of telomeres is an indication of completeness of the assembly. These were analysed by searching for the accepted TTAGG consensus sequence for Hymenoptera [34] in TRF analysis output, estimating their distance to the ends of chromosomes, and comparing the results to that of other 2–7 bp repeats, including non-TTAGG 5 bp repeats. Results (Fig. 2) showed that TTAGG were repeated with at least 842 copies when present at the extremities of chromosomes, whereas other interstitial TTAGG repeats had only 117 repeats or less (mean = 21.3, median = 16.7), a size distribution close to that of other pentanucleotide repeats (mean = 24.2, median = 14.4). See also Additional file 1 Figure S8 and Additional file 2 Tables S6, S7 and S8 for data on other STR motifs. In the AMelMel1.1 assembly, no TTAGG repeats were found on chromosomes 3, 7, 12, and 15, and they were found only at the beginning of chromosome 1. In contrast, in the HAv3.1 assembly, they could be found at both extremities of chromosome 1, but were absent from chromosomes 5 and 11 [11]. An AATAT repeat was found at the beginning of chromosome 15 in our assembly.

The *Alu*I and *Ava*I repetitive sequences, which were previously described as being respectively telomeric and centromeric [35], were localised on the AMelMel1.1 assembly by BLAST search and the number of copies per locus detected was counted (Fig. 2). The *Alu*I repeat was found at the start of chromosomes 2 (6 repeats), 7 (3 repeats), 11 (46 repeats), and 12 (32 repeats). In addition, a single *Alu*I element was found around position 8 Mb on chromosome 15, at more than 1.5 Mb from the distal end. Curiously, the *Alu*I repeats found on chromosomes 2 and 11 were at the opposite end from the TTAGG sequences we detected (Fig. 2). The *Ava*I repeat was found as arrays at single loci on chromosomes 1, 2, 4, 9, 11, and 14. Only 4 copies in the array were found on chromosome 1, the other arrays having between 10 and more than 30 copies. The *Ava*I repeats were at the start of chromosomes 9 and 14, at the opposite end from the TTAGG repeats. On the other four chromosomes, they were at least at 1.8 Mb from a chromosome end (Fig. 2).

### Recombination pattern

**Fig. 3:**
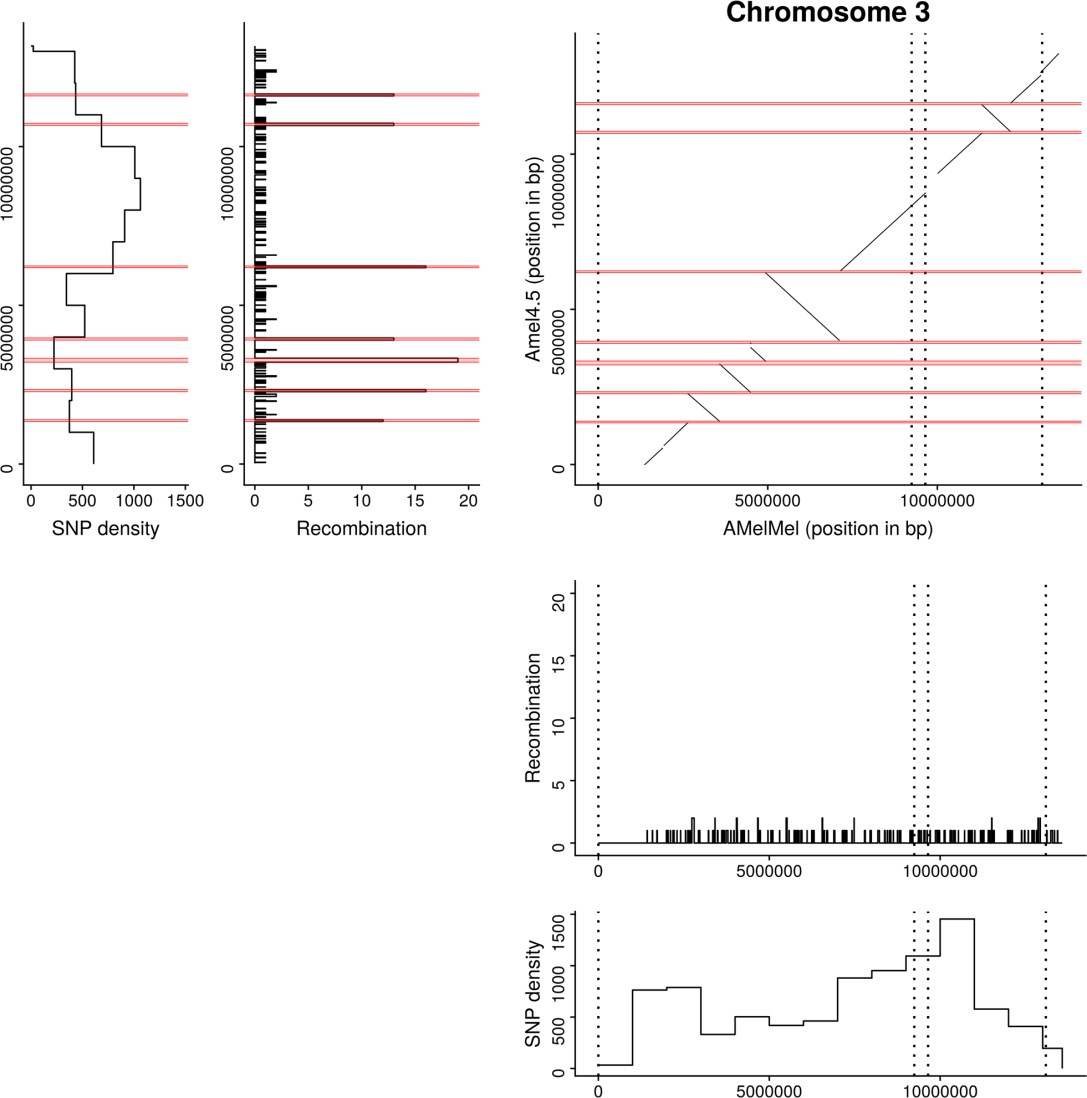
Comparison of Amel4.5 and AMelMel1.1 assemblies for chromosome 3. Abscissa: AMelMel. Ordinate: Amel4.5. AMelMel contig borders are represented with vertical dotted lines. In addition, for both Amel4.5 and AMelMel, the position and number of recombination events detected along the chromosome are represented for each interval flanked by informative markers in the meioses analyzed. Average SNP density and recombination rate are given for 1 Mb windows. Regions indicated in red on the Amel4.5 assembly represent recombination ‘hotspots’ regions, where the number of recombination events between two informative SNPs is higher than five. See supplementary data for the other chromosomes

Having used cross-over detection and a genetic map for contig scaffolding, we could estimate the total genetic map for AMelMel, which was approximately 50 Morgans long, giving an average recombination rate in the genome of 23 cM/Mb, close to the first estimates based on RAPD and microsatellite genetic maps [36–39] and to the most recent estimates based on SNPs [11, 40] (Table 1). However, although we used the same sequencing dataset as in Liu et al. [16], we found a drastic reduction in recombination rate between our genetic map and the one they initially published, which was 37 cM/Mb (Table 1). A big difference is that the latter was based on alignments of sequence reads to Amel4.5. When aligning our assembly to Amel4.5, we find an agreement on the chromosomal assignment of the contigs, but reveal many discrepancies in the orientation of large chromosome segments. At most breakpoint positions between the two assemblies, recombination hotspots were detected on Amel4.5 (Fig. 3 and see Additional file 4 Figure S23), suggesting these assembly errors were responsible for the overall higher recombination rate observed in Liu et al. [16]. This reduction from 37 to 23 cM/Mb is explained by these artefactual recombination hotspots detected in Amel4.5 at the breakpoint positions where the two assemblies disagree, that are absent in AMelMel1.1 (i.e. for chromosome 3 shown in Fig. 3 and see Additional file 4 Figure S23 for all the chromosomes).

**Table 1:** Literature comparison of *Apis mellifera* genetic maps

	Data	Physical size (Mb)	Genetic size (M)	CO/chromosome*	cM/Mb
Hunt and Page [36]	Microsatellites	178	34.5	4.3	19.4
Solignac et al. [37]	Microsatellites	178	40.6	–	22.8
Solignac et al. [38]	Microsatellites	186	40	–	22.04
Beye et al. [39]	Microsatellites	238	45.5	5.7	19
Liu et al. [16]	SNP	220	81.4	5.1	37
Wallberg et al. [40]	SNP	229	59.5	–	26
Wallberg et al. [11]	SNP	219	47.3	–	21.6
AMelMel1.1	SNP	220	50	3.1	23

*Number of cross-overs per chromosome

### High conservation of tandem repeat sequences across chromosomes

We used TRF to further localise and analyse repeat arrays in the whole honey bee genome. Interestingly, two major period size classes for tandem repeats could be found: one in the size range of 91–93 bp, with a maximum number of 231 repeats, hereafter called the 91 bp repeat, and the second in the size range of 367–371 bp, with a maximum number of 100 repeats, called the 371 bp repeat (see Additional file 1 Figure S9). The 91 repeats were found on all chromosomes, whereas the 371 bp repeats were on all chromosomes except chromosome 16 (see Additional file 1 Figure S10). Interestingly, very long repeats whose length is within the range of the sequence reads, were often found at the junction between two sequence contigs, confirming they could be responsible for the impossibility to sequence and assemble these regions properly (see Fig. 2, see Additional file 1 Figure S11).

**Fig. 4:**
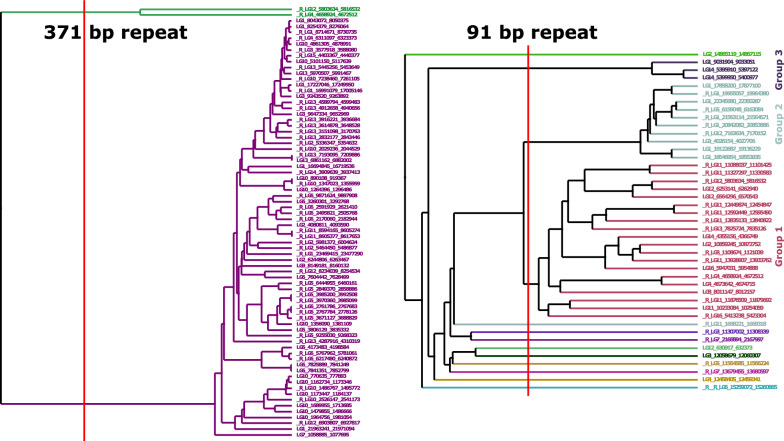
Phylogenetic trees for the tandem repeats of period size 91–93 and 367–371 bp. Only tandem repeats with ten or more elements, such as detected by Tandem Repeat Finder, were considered. Left: phylogenetic tree for the 74 sequences with a period size of 367–371 bp. Right: phylogenetic tree for the 43 sequences with a period size of 91–93 bp. The vertical red lines indicate the cut-off that was used to define the groups of sequence based on similarity

We further investigated the nature of the 91 and 371 repeats by analysing their potential homogeneity in terms of sequence content. Summary statistics for the two classes showed very different distributions in terms of repeat copy numbers within tandem arrays (see Additional file 1 Figure S12 and see Additional file 2 Table S9). There was a total of 345 arrays of the 91 bp repeat in the genome and 131 arrays of the 371 bp repeats. However, these numbers dropped to 43 and 74 respectively when only considering tandem arrays of more than 10 repeats, suggesting that most of the 91 bp repeats had less than 10 elements (see Additional file 1 Figure S12). To investigate sequence homogeneity within each of the two repeat classes, we selected the repeat sequence defined by TRF for repeats that had strictly more than ten copies in tandem within an array. For the 91 bp repeat, we selected for 91 ≤ period size ≤ 93 and for the 371 bp repeat 367 ≤ period size ≤ 371, as suggested by the graph shown in Additional file 1 Figure S9. Then, for each repeat class, we performed a multi-sequence alignment with MAFFT, and produced an average distance tree with Jalview [29]. Results showed that out of the 74 sequences of the 371 bp repeat class, 72 were clearly grouped together, having high similarity (Fig. 4), whereas the 43 sequences of the 91 bp repeat class showed lower similarity. We therefore decided to subdivide the 91 bp repeat class into three groups of 20, 10, and 3 sequences, based on the average distance tree (Fig. 4). The remaining ten 91 bp repeat class sequences were singletons. A consensus sequence was made for each of the four group of sequences and used for a BLAST search in the AMelMel1.1 assembly. Homogeneity of the 371 bp consensus sequence was confirmed by the detection of a very high number of hits of high similarity that covered the overall length of the queries (see Additional file 1 Figure S13). In contrast, for the three different consensus sequences that were used separately for the 91 bp repeat, alignment length and sequence similarities were lower, confirming that this repeat is defined more by its size, rather than by a specific repeat family based on sequence composition (see Additional file 1 Figure S13).

We then searched for the possible presence of the 371 and 91 bp repeats in other organisms. BLAST searches with each of the 371 bp repeat consensus sequences did not find any significant hit in the NCBI nucleic collection database. When searching with each of the 91 bp consensus repeats, four hits were found: three consensus sequences from repeat arrays from chromosome 11 and one consensus sequences from a repeat array from chromosome 12 showed sequence similarity to fragments of predicted lncRNAs *LOC116185390*, *LOC105734921*, *LOC116415009* and *LOC116185696*, from unknown scaffolds of the genome assemblies of *Apis dorsata* and *Apis florea*. However, these lncRNAs are composed of two exons and span close to 1.5 kb in the genomes of *Apis dorsata* and *Apis florea*, suggesting that the 91 bp sequences correspond to only a portion (one out of two exons) of these lncRNAs. To investigate further, we performed BLAST searches with each of the consensus sequences directly on the refseq_genomes databases of *Apis cerana*, *Apis dorsata,* and *Apis florea* and a very high number of hits were found, suggesting that the 371 bp and 91 bp repeats were also present in these three genomes, with an apparent slightly higher percent identity for the 91 bp repeat (see Additional file 1 Figure S14).

### Difference in the number of repeats of 5S ribosomal RNA genes

**Fig. 5:**
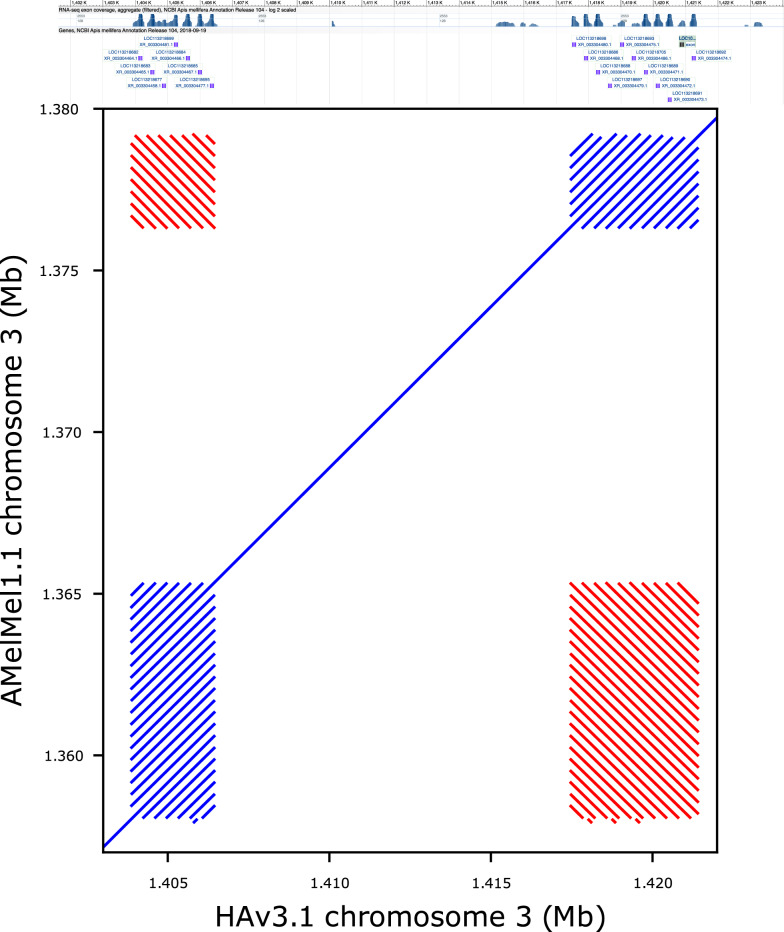
Differences in copy numbers for 5S RNA ribosomal genes. Shown are two of the loci containing 5S RNA genes that are present at 15 kb distance on chromosome 3. Top: screenshot of the NCBI genome viewer for the region showing the annotation for the 5S RNA genes. Bottom: dotplot alignment of HAv3.1 (x-axis) and AMelMel1.1 (y-axis) in the region. The first group of genes in the bottom left contains seven genes in HAv3.1 and twenty in AMelMel1.1 on the forward strand. The second in the top right contains eleven genes in HAv3.1 and eight in AMelMel1.1 on the reverse strand. The red lines off diagonal show the sequence similarity between the two groups of genes and confirm that the two gene clusters are in reverse orientation

Genes that are repeated in tandem can often vary in numbers between individuals through unequal cross-overs [41] and are, therefore, good candidates to study functional variation related to large rearrangements. A typical example of such genes is the 5S ribosomal RNA genes, whose copy number can vary greatly in the genome [42–44]. Alignment of a region from the AMelMel1.1 and HAv3.1 assemblies in a region on chromosome 3 that contained 5S ribosomal RNA genes, showed variation in the number of these genes between the two genomes (Fig. 5.). The period size of one of the repeat arrays of 5S genes was 357 bp, while that of the other was 373 bp. However, inclusion of this sequence in the multiple sequence analysis of the 371 bp repeat showed that these two sequences are different (see Additional file 1 Figure S15).

### Inversions and indels between AMelMel1.1 and HAv3.1

**Fig. 6:**
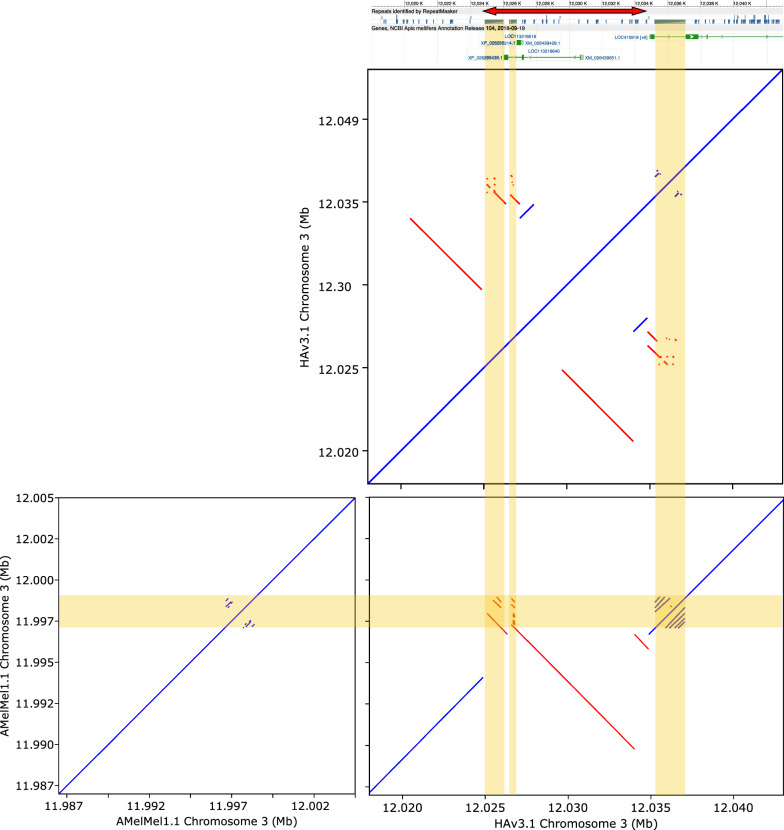
A 10 kb inverted duplication on chromosome 3 between HAv3.1 and AMelMel1.1. Bottom right: a dot plot representation of the alignment with LAST of AMelMel1.1 to HAv3.1 shows a 10 kb inversion on chromosome 3. Self-alignments of AMelMel1.1 (left) and HAv3.1 (top) show that the latter has an inverted repeated sequence in the region. The vertical yellow lines show the position of repeats that were previously detected and shown in the NCBI annotation (grey boxes) and that were also found in our LAST alignments. NCBI annotation of genes are in green

One-to-one split alignments produced by aligning AMelMel1.1 on HAv3.1 with LAST were used to detect inversions larger than 1000 bp between the two genomes. The largest inversion detected was on chromosome 7 and was larger than 1.6 Mb (see Additional file 3 Figure S22 and see Additional file 5 Figure S24). It should be noted, that a similar rearrangement on chromosome 7 was previously detected when comparing a genome assembly of an *A. m. ligustica* samples with the HAv3.1 reference [45]. Although close to one hundred other inversions could be detected, their visual inspection on dotplot graphs showed that 53 were within complex repeat patterns that were present at the junction between contigs, 32 within other complex repeat elements, and only 12 were in the middle of the high-quality sequence contigs in both assemblies, thus representing well supported inversions. Apart the large inversion on chromosome 7, the smallest was 1055 bp long and the largest 25,608 bp long (see Additional file 2 Table S10 and see Additional file 5 Figure S24). Interestingly, some inversions are in genes and can involve repeat elements found in both assemblies. In the example shown in Fig. 6, a local inverted duplicated region that was seen in the HAv3.1 assembly was absent in AMelMel1.1. This chromosomal segment contains a portion of the gene model *LOC113218640*, which has no direct annotation in the HAv3.1 assembly, but is described as coding for a *bric-a-brac 1-like* protein. *Bric-a-brac* was shown to be involved in body pigmentation in Drosophila [46]. Another interesting inversion is 11 kb long on chromosome 3, in an intron of *Rhomboid*, a gene involved in the formation of wing veins in Drosophila [47]. A more complex rearrangement involves a gene labelled as a probable nuclear hormone receptor, *HR38*, which is involved in synchronizing the reproductive activity in *Agrotis ipsilon* [48] and in the larval-pupal transition in *Leptinotarsa decemlineata* [49]. Other genes involved in the inversions described have various functions [50–55] and are reported in Additional file 2 Table S10.

The vcf file produced by SVIM-asm was used to study the size distribution of indels that were larger than 40 bp. In total, 9589 indels were detected, only 66 of which were larger than 1000 bp and six larger than 10 kb (see Additional file 1 Figure S16). The largest insertion of a segment that was present in AMelMel1.1 and absent in HAv3.1 that was detected was 31,640 bp long and the largest segment that was present in HAv3.1 and absent in AMelMel1.1 was 22,461 bp long. Two hundred and ninety-one indels overlapped totally or partially with exons, 289 of which were smaller than 725 bp (see Additional file 1 Figure S17). One insertion 17,759 bp long, specific to AMelMel, was located in exon 3 of the uncharacterised gene *LOC410644* and one 6,953 bp deletion on chromosome 10 removes exon 2 from the uncharacterised gene *LOC100577772* in AMelMel.

### Using both assemblies for analysis of two medium-size indels in honey bee subspecies

**Fig. 7:**
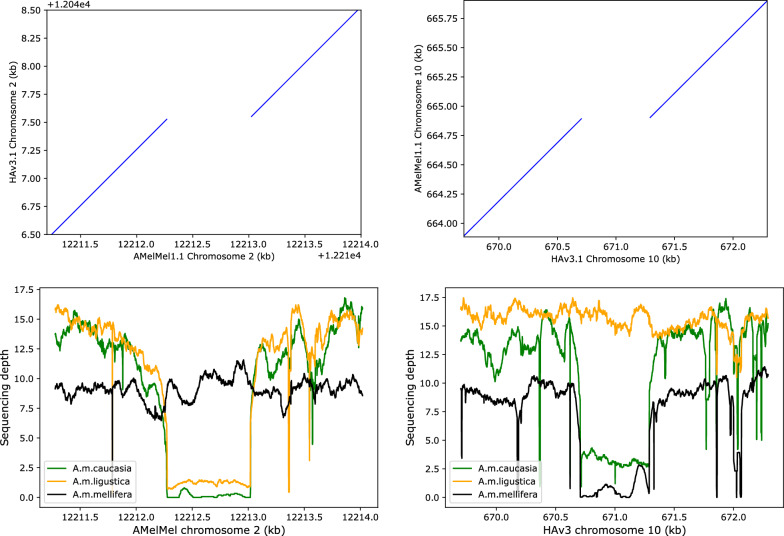
Insertions and deletions in *Apis mellifera* subspecies. Analysis of nuclear mitochondrial DNA (NUMT) insertions detected in only one assembly. Top: dotplot representation of LAST alignments between the two assemblies show a 745 bp variant present in AMelMel1.1 on chromosome 2 and absent in HAv3.1 (left) and a 576 bp variant present in HAv3.1 chromosome 10 and absent in AMelMel1.1 (right). For each variant, sequencing depths were evaluated for individual samples on the reference in which it is present. Bottom: mean sequencing depth per subspecies, with *A. m. caucasia* (15 samples) in green, *A. m. ligustica* (30 samples) in yellow and *A. m. mellifera* (35 samples) in black. Results suggest that most of the *A. m. mellifera* samples contain the insertion present in the AMelMel1.1 assembly on chromosome 2, as the sequencing depth remains constant throughout the region, and not the one present in the HAv3.1 assembly on chromosome 10, as indicated by a sequencing depth close to zero. Inversely, most of the *A. m. ligustica* samples contain the insertion present in the HAv3.1 assembly on chromosome 10 and not the one in the AMelMel1.1 assembly on chromosome 2. Most *A. m. caucasia* samples lack the insertion present in the AMelMel1.1 assembly and a few seem to have the insertion present in the HAv3.1 assembly

**Fig. 8:**
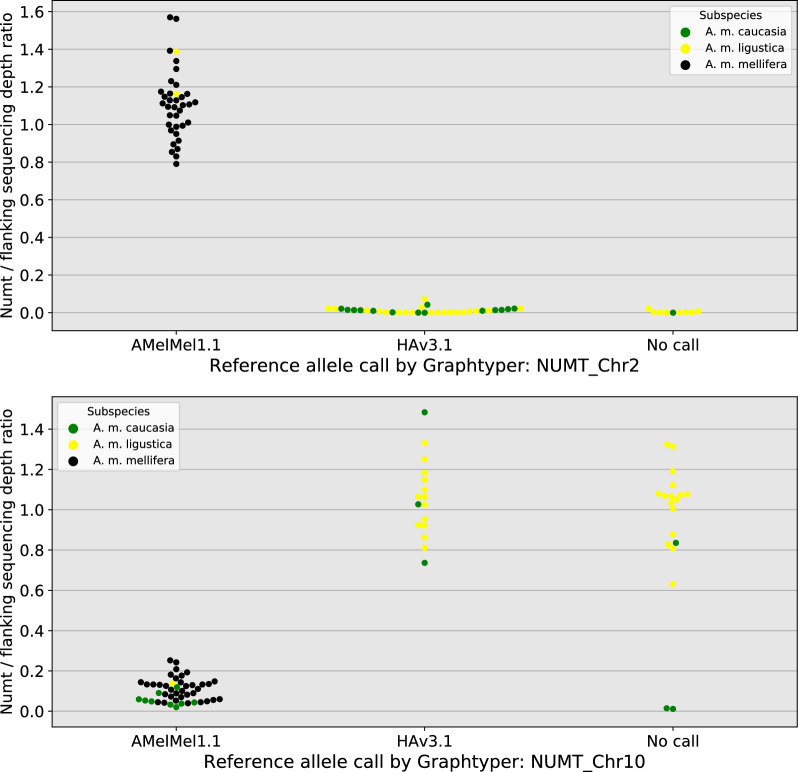
Comparing the indel variant calling between sequencing depth analysis and Graphtyper2. Presence or absence of the NUMTs in the samples was evaluated by the pangenome graph approach with Graphtyper2 (x-axis) and by estimating the sequencing depth at the position of the NUMTs on the genome in which they are present (y-axis). Sequencing depths were normalised by calculating the ratio between sequencing depth at the position of the NUMT sequence and that of the flanking sequence. Nine out of 80 samples (11%) could not be called for NUMT_Chr2 and 19 (24%) not for NUMT_Chr10. When alleles could be called by Graphtyper2, results agreed with the data based on sequencing depth

To demonstrate the utility of using two reference genomes for analysing structural variants, we studied two indels corresponding to nuclear mitochondrial DNA (NUMT), that were detected by using minimap2 [30] and SVIM-asm [31]. The first, NUMT_Chr2, is 745 bp long, has 92.7% identity over 99% of its length to HAv3.1 mitochondrial DNA, is present in the AMelMel1.1 assembly on chromosome 2 at positions 12,212,275–12,213,020, and is absent from the HAv3.1 assembly. The second, NUMT_Chr10, is 576 bp long, has 92.5% identity over 94% of its length to HAv3.1 mitochondrial DNA, is present in the HAv3.1 assembly on chromosome 10 at positions 670,675–671,251, and is absent in the AMelMel1.1 assembly. Presence and absence of these two NUMTs were tested in three honey bee subspecies: *A. m. mellifera* (n = 35), *A. m. ligustica* (n = 30) and *A. m. caucasia* (n = 15), for which Illumina sequencing data was aligned to both reference genomes. Inspection of mean sequencing depth over all 80 samples in the regions of NUMT_Chr2 and NUMT_Chr10 indicated a decrease of the mean depth and an increase of its variance (see Additional file 1 Figure S18), suggesting the existence of a presence / absence polymorphism. When inspecting the sequencing depth per population, the *A. m. mellifera* samples showed a constant value over NUMT_Chr2 and a depth close to zero over NUMT_Chr10, whereas the *A. m. ligustica* show an inverse tendency (Fig. 7). The *A. m. caucasia* samples did not appear to have NUMT_Chr2 in their genomes, whereas a few may have NUMT_Chr10, as suggested by the incomplete drop of mean sequencing depth on HAv3.1 in the corresponding region (Fig. 7). To genotype our samples individually, we used two methods. The first was to estimate individual sequencing depth in the chromosomal region that delimits the NUMTs, by using AMelMel1.1 as reference genome for NUMT_Chr2 and HAv3.1 for NUMT_Chr10 (see methods). All 80 samples could thereafter be called unambiguously and assigned to one of two groups (presence or absence) by K-means clustering (see Additional file 1 Figure S19). The second method tested was to use GraphTyper2 [33], allowing the genotyping of structural variation using pangenome graphs. Our GraphTyper2 results showed that the calling of samples was incomplete, with a high proportion of no-calls, and that using individual bam files of alignments to one or to the other reference genome can greatly influence the call rate (see Additional file 2 Table S11). Indeed, for detection of variants with minimap2 and SVIM-asm, a reference genome must be specified and bam files of alignments to this specific reference genome must be used to perform individual genotyping. So, we first used HAv3.1 as reference, resulting in a genotyping call rate of 78.7% for NUMT_Chr2 and null for NUMT_Chr10, with no result in the output file from GraphTyper2 at all. To check if the reference genome could influence the results, we also performed the analysis by using AMelMel1.1 as reference and this time the call rate was 85.0% for NUMT_Chr2, and 76.2% for NUMT_Chr10. When genotype calls were successfully obtained in both analyses, results were identical and also concordant with the analysis based on sequencing depth, showing that results were consistent when genotyping was possible with Graphtyper2. Two samples were called as heterozygotes for NUMT_Chr2 when using AMelMel1.1 as reference and were counted as “no calls” because our samples were haploid. Low sequencing depth could have been a possible explanation for the absence of genotyping results with GraphTyper2 in some of the samples, but this does not seem to be the case, as all samples that failed genotyping had at least 8X average sequencing depth in the sequence flanking the NUMTs analysed, while successful genotyping could be obtained for samples having as little as 2X sequencing depth (see Additional file 1 Figure S20). Importantly, the individual genotyping results confirmed the overall impression that the presence or absence of the NUMT insertions are specific to the subspecies analysed, with most, if not all samples having identical within-population genotypes, except for NUMT_Chr10 in *A. m. caucasia*, for which four out of eleven samples had a different allele. Interestingly, NUMT_Chr2 is present in all *A. m. mellifera* and in only two *A. m. ligustica* samples, and absent from all other samples, while NUMT_Chr10 is absent from *A. m. mellifera* samples and present in all but one *A. m. ligustica* samples and in four *A. m. caucasia* samples (Figs. 7, 8).

### Difference in sequencing depth according to the genome used for alignment

Overall alignment depths of sequence reads on both the HAv3.1 and AMelMel reference genome assemblies were determined for all 80 samples studied. The ratios between the alignment depths on each of the two genomes show that *A. m. caucasia* and *A. m. ligustica* samples had a slightly higher sequencing depth on HAv3.1 than on AMelMel1.1 and inversely, *A. m. mellifera* samples had a slightly higher sequencing depth on AMelMel1.1 than on HAv3.1 (see Additional file 1 Figure S21).

## Discussion

### AMelMel assembly quality and comparison to other honey bee assemblies

Although five chromosome level genome assemblies for *Apis mellifera* are available [56], ours has the originality of representing *Apis mellifera mellifera*. Indeed, this subspecies is genetically distinct from *Apis mellifera ligustica*, *Apis mellifera carnica,* and *Apis mellifera caucasia* [6] which are represented by the four other assemblies. To ensure that the sample used is a thorough representative of the *A. m. mellifera* genetic background, our genome assembly relied on a single drone from a black honey bee conservation population that has been kept in isolation on a small island since 1991, thus preventing admixture. While this ensures at best that the AMelMel1.1 assembly is a true representative of the *A. m. mellifera* genetic ancestry, other populations from this subspecies in other geographic regions and also isolated for conservation purposes, may have diverged slightly from the sequenced individual. Future work should extend the repertoire of *A. m. mellifera* variation through long-read sequencing of samples from other origins. Another originality of our study is that the contigs we obtained were scaffolded into chromosomes using a genetic (recombination) map rather than the now more common HiC chromatin conformation and Bionano optical maps methods [57, 58]. Compared to the current HAv3.1 reference genome [11], our assembly is slightly longer (227 Mb versus 225 Mb) and was built from a lower number of contigs (200 versus 228) with very similar N50 contig values (5.1 Mb versus 5.4 Mb). However, the overall final coverage was slightly smaller (137 X Pac Bio and Illumina reads in AMelMel1.1 versus 192 X in HAv3.1). The BUSCO statistics were also very similar between AMelMel1.1 and HAv3.1 because the contig building was based on PacBio reads in both cases, with some correction using Illumina reads. In only one case did assembly of contigs into chromosomes using the recombination data fail to accurately order and orient a contig, a large contig on chromosome seven. Despite this limitation, we were able to orient this contig thanks to careful analysis of tandem repeat elements at its boundaries. Sequencing data for both HAv3.1 and our assembly, AmelMel1.1, are from a single haploid drone, which is a tremendous advantage for the resolution of regions that are largely composed of repeat elements. This was recently demonstrated in the human Telomere-to-Telomere project, for which a complete hydatidiform mole haploid cell line was used, which helped solve complex structures such as centromeres [59]. Our results show however that, although sequencing of repeat elements and especially of challenging tandem repeats seems to be resolved by the use of a single haploid sample and long reads, there are cases in which the total length of monotonous repeats is larger than the read lengths, preventing local assembly. As a result, for almost all contig boundaries investigated, long stretches of tandem repeats were found (Fig. 2). Interestingly, chromosome 16, which was obtained as a single contig, had no stretch of tandem repeats that exceeded 10 kb.

### Genetic maps and recombination rate in the honey bee

Having used genetic recombination data to scaffold our contigs, we were able to build a new recombination map and estimate 23 cM/Mbp for the overall recombination rate in the honey bee [16], which is of the same magnitude as the latest values from [11] and also congruent with prior values [38–40] (Table 1). It is interesting to note that the public sequencing dataset that we used, representing 43 drone genome offspring of three queens, gave a much higher estimate of 37 cM/Mb when it was previously used to generate genotyping data by alignment to the Amel4.5 reference genome [16]. On closer inspection, this higher overall recombination rate in Liu et al. [16] was due to very specific false recombination hotspots that appeared at contig junctions in Amel4.5, when at least one of them was inverted compared to AMelMel1.1 (Fig. 3 and see Additional file 4 Figure S23). This illustrates the importance of the quality of the reference genome for such studies. Errors in local estimates of recombination rates when using a mis-assembled reference genome will in turn affect any analysis based on recombination maps or that include linkage disequilibrium.

### Tandem repeats and current limits for obtaining chromosome-wide contigs

We found a high occurrence of conserved tandem repeats in the honey bee genome, whose lengths and sequence conservation caused problems for scaffolding contigs into chromosomes, the ultimate goal being for each chromosome to be covered by a single contig. Indeed, long stretches of such repeats were found at the boundaries between contigs. Luckily, the only large contig in the assembly that could be placed on chromosome 7 but not oriented due to lack of sufficient genetic data, had different tandem repeats at each of its extremities, allowing us to decide on a correct orientation. However, other regions may still be problematic, the most striking example being the region between 1 and 3 Mb on chromosome 10. In this region, the contigs were small (< 0.2 Mb) due to a high occurrence of tandem repeats, leading to difficulties in their ordering along the chromosome and their orientation. Moreover, these repeats appeared to mostly belong to the highly conserved 371 bp family, preventing their use for contig mapping. This portion of chromosome 10 has also been described as difficult to assemble in other studies [60].

### General chromosome structure: telomeres and centromeres

Cytogenetic studies based on fluorescent in situ hybridization of *Alu*I and *Ava*I probes suggest that the honey bee genome is composed of one large metacentric and 15 acrocentric chromosomes [35]. This is to date still considered as the standard honey bee karyotype structure [11, 34]. However, other data could question this structure, for instance the suggested positions of the centromeres based on sequence characteristics of the HAv3.1 genome assembly, such as the (GC) content and the presence of *Alu*I and *Ava*I repeats on chromosomes 7, 8, and 11 in Wallberg et al. [11].

Regarding telomeres, we were not able to identify the TTAGG consensus sequences on all 17 chromosome ends (two for the metacentric chromosome 1 and one for each of the other fifteen acrocentric chromosomes) where they were expected: none were detected on the right arm of chromosome 1 and on chromosomes 3, 12, and 15. Interestingly, some chromosomes also lacked TTAGG repeats in the HAv3.1 assembly, but these were not the same as in ours (chromosomes 5 and 11). These discrepancies can be due to problems in the assembly of these repeat regions, either due to variations in sequence quality between the two datasets or to local variations in repeat content, rendering the assembly to be of varying difficulty due to biological reasons. It is interesting to note that in the older assemblies of the bee genome, based on the same DH4 strain as used for HAv3.1, extended analyses of telomeric and subtelomeric repeats showed that some chromosomes were easier to analyse than others and that no TTAGG repeats were identified for chromosome 5 [34]. Taken together, although the current sequencing data supports the consensus karyotype structure, we didn’t find that the *Alu*I repeat elements [35] could be considered as a marker of telomeres, because when such repeats were detected at the extremity of a chromosome, this was at the opposite end from the TTAGG repeats (see specifically chromosome 11 in Fig. 2).

The question of the exact position of the centromeres is a more complex one: the centromeres are expected to be at the middle of chromosome 1 and at the proximal end of each of the other chromosomes. The *Ava*I repeat element, which is considered as a marker of centromeres [35], was not found on all chromosomes and even when found, the number of repeats in the array could be as small as four, such as on chromosome 1 (Fig. 2). With the exception of chromosome 11, for which an *Ava*I repeat was found at position 5 Mb, the *Ava*I elements, when they were detected on a chromosome, were found within 2.5 Mb of the chromosome ends, reflecting the results found for HAv3.1 [11]. However, although the positions of the *Ava*I repeats was identical between the two assemblies, the number of repeat elements varied for each given position. Thus, for the moment, the exact position of the centromeres remains uncertain, but the presence of an *Ava*I element remains a plausible indication, especially as these seem to be coincident with other specific characteristics, such as low (GC) content [40] or low levels of polymorphism and recombination rates [6]. If these characteristics are indicators of centromere positions, then chromosome 11 and perhaps also chromosome 7 should be considered sub-metacentric, although then TTAGG repeats would be expected at both of the extremities of these chromosomes, which is not the case in any of the studies to date. Further improvements in genome sequencing and assembly and in obtaining higher-resolution cytogenetic metaphase chromosome preparations will be necessary to elucidate this question.

### Comparing the genomes of two honey bee subspecies

The HAv3.1 assembly is based on a sample from the DH4 line, which is thought to be mainly of *A. m. ligustica* descent [9]. The comparison with our *Apis mellifera mellifera* AMelMel1.1 assembly allows for the detection of rearrangements that occurred between these two distinct genetic types that cannot be detected by short read sequencing. Short sequence fragments repeated in tandem, such as the 91 bp and 371 bp repeats described here, tend to vary in copy number through non-allelic homologous recombination (NAHR) or unequal cross-overs [61]. A rapid observation of the LAST alignment data between the two assemblies suggests that the 371 bp repeat element can vary greatly in copy number and the 91 bp element to a much lesser extent, although these preliminary observations will require more thorough analyses. To date, no obvious function was found for these elements, except for the fact that a BLAST search found that the 91 bp element shows similarity in sequence to one out of two exons of *Apis dorsata* and *Apis florea* lncRNAs, suggesting that these are incomplete and consequently not active in the repeat arrays. However, annotation of the lncRNAs in *Apis dorsata* and *Apis florea* is only based on alignment of short read RNA-seq data. More work is needed to confirm this finding concerning the 91 bp repeat and further comparisons with other bee genomes whose sequences are underway [62] will help understand these interesting genome elements.

The 5S ribosomal RNA genes are another interesting case of variation in gene number and studies in mouse and human have shown that this variation may be important for a balanced dosage of rRNA that can have implications in diseases [37, 38]. It would be interesting to determine whether the variations in 5S gene numbers observed here is a difference between the two honey bee subspecies investigated or if intra-population variation can be found.

After screening out rearrangements that could be due to errors associated with assembly problems, such as inversions of complete small contigs, thirteen inversions larger than 1 kb were detected between the two genomes. Of these, a large 1.6 Mb inversion on chromosome 7 is likely an error in HAv3.1, as it was also seen in the sequence of a closely related sample from the *Apis mellifera ligustica* subspecies [45]. Of the twelve remaining inversions, some involve genes that are present either at one of the breakpoints and that have inversions within their structure (usually introns) or whose structure remains intact but are in reverse orientation. Eventually, interesting functions that may explain some of the phenotypic differences found between the two subspecies represented by our dataset will be found (see Additional file 2 Table S10). Even when restricting to genes for which functions have been observed in insects, three genes stand out. One is *Bric-a-brac 1-like*, whose implication in body pigmentation in Drosophila [46], could be linked to our two reference genomes, representing light (yellow) and dark coloured honey bee subspecies. Another is *Rhomboid*, which has previously been shown to be involved in the formation of wing veins in Drosophila [47]. A third is the hormone receptor *HR38*, which has been shown to be involved in synchronisation of reproductive activity in the moth *Agrotis ipsilon* and in larval-pupal transition in the Colorado potato beetle *Leptinotarsa decemlineata* [43, 44].

The number of insertions and deletions between the two assemblies detected by our approach was less than 10,000, only very few (66) were larger than 1000 bp, and fewer than 300 overlapped with annotated exons. The importance of these findings will have to be investigated in more detail, first by confirmation by long-read sequencing of additional samples, and also by improving the annotation of the assemblies, in which gene models may be imperfect, especially for non-coding genes.

### Perspectives for pangenomics and the test case of nuclear mitochondrial DNA segments

The limitations associated with the use of a single reference genome for an exhaustive analysis of all possible samples from a population has long been documented [63]. One of the most important limitations is the fact that sequence segments, often containing genes, can be specific to a limited number of individuals from a species or population. As a consequence, sequencing reads from such sequence segments that are present in a sample but absent in the reference, will at best be discarded or will map to wrong positions on the reference, thus creating false variants. Just by comparing two *A. mellifera* genome assemblies, we show here that large insertions and deletion can be found, 6 of which were larger than 10 kb. Although only a limited number overlapped with exons, their impact on phenotypes can be considerable through modifications of gene regulation, for instance by inserting, removing, or changing the position of enhancers and other regulatory elements. It is therefore essential that large structural variants be considered in association studies, and therefore be genotyped. Furthermore, loss of information through the use of a single reference genome can also concern SNPs, as in some regions, high densities of variants may lead to low mapping depths, due to a number of mismatches per read that exceed thresholds required for high mapping quality. We have indeed shown here that mapping reads from a given *A. mellifera* subspecies to a reference genome of the same subspecies yields higher sequencing depths than when a reference from a different subspecies is used. This suggests that some information is lost in the process. To address all these issues, the reference genome model can now be extended to graph representations of genomes, in which alternate loci can be represented [64].

To test the potential of a pangenome approach for honey bees, we used the two reference genomes to genotype structural variants, in the form of two NUMTs that were present in either the HAv3.1 or in the AMelMel1.1 assembly. These variants were selected as a test case for presence or absence of a 745 bp fragment in the case of NUMT_Chr2 and a 576 bp fragment for NUMT_Chr10. Since the 80 population samples analysed here were all haploid, each of the NUMTs analysed should be either present or absent in a given individual, avoiding the uncertainties associated with heterozygote status and greatly simplifying the genotype calling. However, despite this, results showed that the Graphtyper2 approach did not succeed in calling genotypes for all samples. Such results may be caused by the fact that Graphtyper2 is biased towards one of these two genomes, although it used a vcf file that represented the alternative paths through the two genomes. This results potentially from the fact that (i) the population data needs to be used as bam files that resulted from a primary mapping to one or the other reference genome and (ii) the vcf file that represents the alternative paths through the genomes contains a standard representation of reference and alternate alleles. This reference-bias became really obvious when HAv3.1 was used as reference for the construction of the vcf and primary mapping of reads, NUMT_Chr10 could not be genotyped at all, while a higher genotype calling rate was obtained when AMelMel1.1 was used as the primary reference. Other methods for building graph genomes and aligning reads to them have been described, such as Minigraph-Cactus for graph construction [65] and Giraffe for the alignment of reads [66]. However, these remain biased, as a ‘reference’ assembly is chosen as an initial backbone, which is then augmented in turn with variation from the remaining assemblies [65]. Recently, new methods such as PanGenome Graph Builder (PGGB) [67] were introduced to address the reference-bias problem and could be tested. Although more exhaustive than single reference approaches, pangenomes are more complex to use in practice, due to specific problems they introduce, such as the definition of genome coordinates, which is essential when comparing different studies [66].

## Conclusions

In conclusion, we have presented a genome assembly for the honey bee *Apis mellifera mellifera* that is from a different subspecies than the current reference genome. One originality of the assembly process was the use of recombination data rather than optical maps or HiC to scaffold contigs into chromosomes. We characterised for the first time long tandem repeats that are present in the genome and that were responsible for most sequence discontinuities and showed that these belong to two main repeats families that yet need to be further characterised and whose potential function in the genome remains to be investigated. Finally, we demonstrated the value of having two reference-quality genomes for the detection of structural variants, such as inversions and insertions-deletions and demonstrated the possibility of using a pangenome approach to genotype such variants in honey bee populations.

### Supplementary Information

Additional file 1. Figure S1. Mortar and potter used for grinding individual drones. Figure S2. A Fragment analyzer results for sample OUE7B. B Fragment analyzer results for sample OUE8B. Figure S3. Distribution of read lengths per SMRTcell. Figure S4. Size distribution of sequencing reads from all 36 SMRTcells. Figure S5. Size distribution of the length of the contigs assembled with Canu. Figure S6. Alignment of contigs to Amel4.5 and detection of SNPs in the data from [16]. Figure S7. Ordering contigs by using crossing-over events. Figure S8. Telomeric repeats and other repeats of small period size. Figure S9. Period size and copy number of tandem repeats. Figure S10. Chromosome distribution of the 91 bp repeats and the 371 bp repeat. Figure S11. Tandem repeats of period size 1000-2000 bp detected in the AMelMel assembly. Figure S12. Distribution of the number of repeats in tandem arrays for the 91 bp and 371 bp repeats. Figure S13. Summaries from the BLAST hits on AMelMel1.1 obtained with the consensus sequences from the major group for the 371 bp repeats and the three major groups for the 91 bp repeats. Figure S14. BLAST searches with the 371 bp and the 91bp on *Apis* genomes. Figure S15. Phylogenetic trees for the tandem repeats of period size 367-374 bp. Figure S16. Sequencing depth within and around insertions and deletions in *Apis mellifera* subspecies. Figure S17. Sequencing depth and genotype deduction in NUMT_Chr2 and NUMT_Chr10. Figure S18. Sequencing depth and call rate with Graphtyper 2. Figure S19. Sequencing depth and genotype deduction in NUMT_Chr2 and NUMT_Chr10. Figure S20. Sequencing depth and call rate with Graphtyper 2. Figure S21. Overall alignment depth according to the reference used.

Additional file 2. Table S1. Illumina reads downloaded from NCBI SRA project SRP043350. Data from [16]. Table S2. Samples used for analysing the InDel variants. Table S3. Canu assembly statistics. Table S4. Statistics on the BUSCO genes expected to be found in metazoa and hymenoptera. Table S5. Repeats detected by Tandem Repeat Finder at the breakpoints of the 5^th^ contig of chromosome 7. Table S6. Short tandem repeats within 1 kb of chromosome ends. Table S7. Short tandem repeats with > 400 copies. Table S8. Statistics on non-TTAGG Short Tandem Repeats with more than 10 repeat copies. Table S9. Summary statistics for the 91 and 371 bp repeats. Table S10. Inversions detected between AMelMel1.1 and HAv3.1. Table S11. Genotypes of NUMT_Chr2 and NUMT_Chr10 with Graphtyper and by examining local sequencing depth.

Additional file 3. Figure S22. Comparison of HAv3.1 and AMelMel1.1 genome assemblies. Each subpanel represents one of the 16 chromosomes of the honey bee.

Additional file 4. Figure S23. Comparison of Amel4.5 and AMelMel1.1 assemblies for each of the 16 chromosomes of the honey bee.

Additional file 5. Figure S24. Inversions larger than 1 kb detected between the AMelMel1.1 and HAv3.1 genome assemblies.

## Data Availability

The AMelMel1.1 assembly has been deposited on the NCBI under the accession number GCA_003314205. The reads of the 36 corresponding PACBIO_SMRT runs are in SRA under the accessions SRR9587836 to SRR9593684. Scripts and supplementary description of bioinformatic analyses are available in GitHub: https://github.com/avignal5/PacificBee/tree/main. Annotation files for the genome assembly are available at https://zenodo.org/records/10459067 for download.
