# Supplementary material for: The black honey bee genome: insights on specific structural elements and a first step towards pangenomes
Source: Genet Sel Evol. 2024 Jun 28;56:51. doi: 10.1186/s12711-024-00917-3 (PMC11212449; doi:10.1186/s12711-024-00917-3)
Supplement: Supplementary file 3 — Additional file 3. Figure S22. Comparison of HAv3.1 and AMelMel1.1 genome assemblies. Each subpanel represents one of the 16 chromosomes of the honey bee. [file 12711_2024_917_MOESM3_ESM.docx]

Additional file 3

**Figure S22: Comparison of HAv3.1 and AMelMel1.1 genome assemblies. Each subpanel represents one of the 16 chromosomes of the honey bee.** Dotplot representation of the alignment of HAv3.1 (x-axis) and AMelMel1.1 (y-axis). Blue: alignments in the same direction, red: alignments in inverted directions. Vertical and horizontal dotted lines represent contig boundaries on the two assemblies.

 **Subpanel 1:** Chromosomes 1 and 2.

 **Subpanel 2:** Chromosomes 3 and 4.

 **Subpanel 3:** Chromosomes 5 and 6. **Subpanel 4:** Chromosomes 7 and 8. **Subpanel 5:** Chromosomes 9 and 10. **Subpanel 6:** Chromosomes 11 and 12. **Subpanel 7:** Chromosomes 13 and 14. **Subpanel 8:** Chromosomes 15 and 16.
