# Supplementary material for: The black honey bee genome: insights on specific structural elements and a first step towards pangenomes
Source: Genet Sel Evol. 2024 Jun 28;56:51. doi: 10.1186/s12711-024-00917-3 (PMC11212449; doi:10.1186/s12711-024-00917-3)
Supplement: Supplementary file 4 — Additional file 4. Figure S23. Comparison of Amel4.5 and AMelMel1.1 assemblies for each of the 16 chromosomes of the honey bee. [file 12711_2024_917_MOESM4_ESM.docx]

Additional file 4

**Figure S23: Comparison of Amel4.5 and AMelMel assemblies for each of the 16 chromosomes of the honey bee.** Abscissa: AMelMel, ordinate: Amel4.5. AMelMel contig borders are represented by vertical dotted lines. Additionally, for both assemblies, the position and number of recombination events detected along the chromosome are represented in each interval flanked by informative markers in the meiosis analysed. Average SNP density, recombination rate and GC% are given for 1Mb windows. Red zones represent recombination ‘hotspots’ regions where number of recombination events between two informative SNPs is higher than five events. Sequencing depth in 1 Mb windows for each of the 3 colonies analyzed to reconstruct the genetic map are in blue.


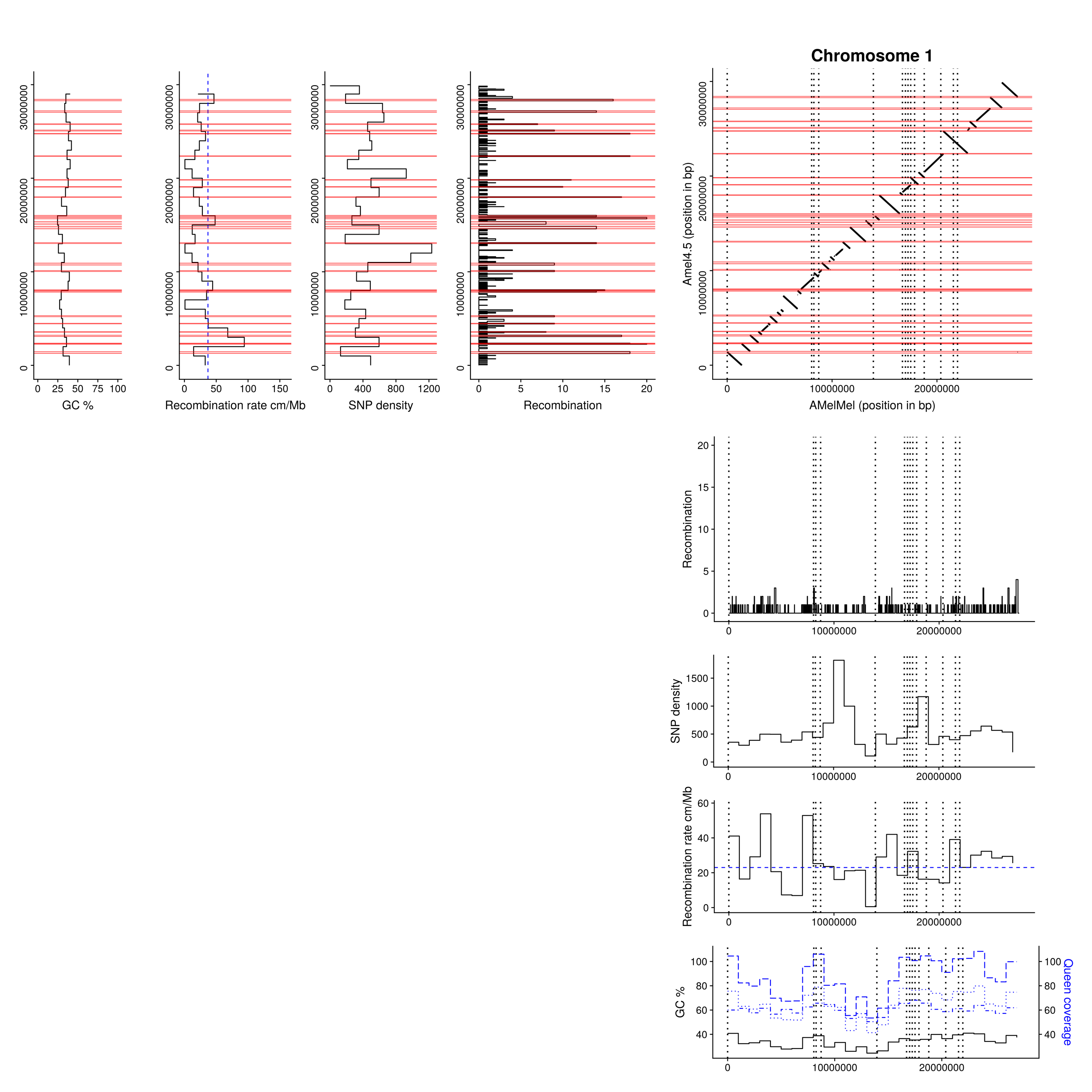


**Subpanel 1:** Chromosome 1


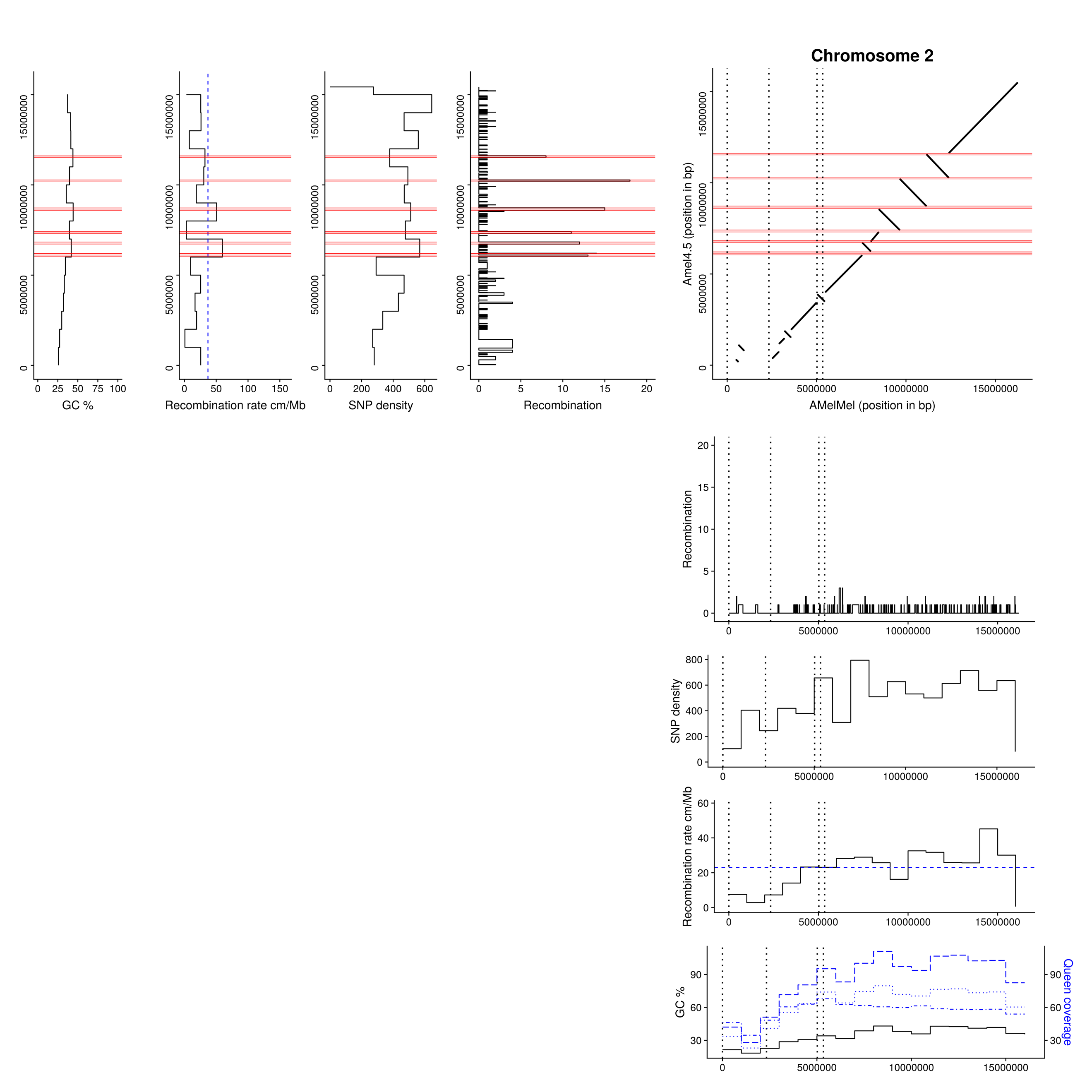


**Subpanel 2:** Chromosome 2.


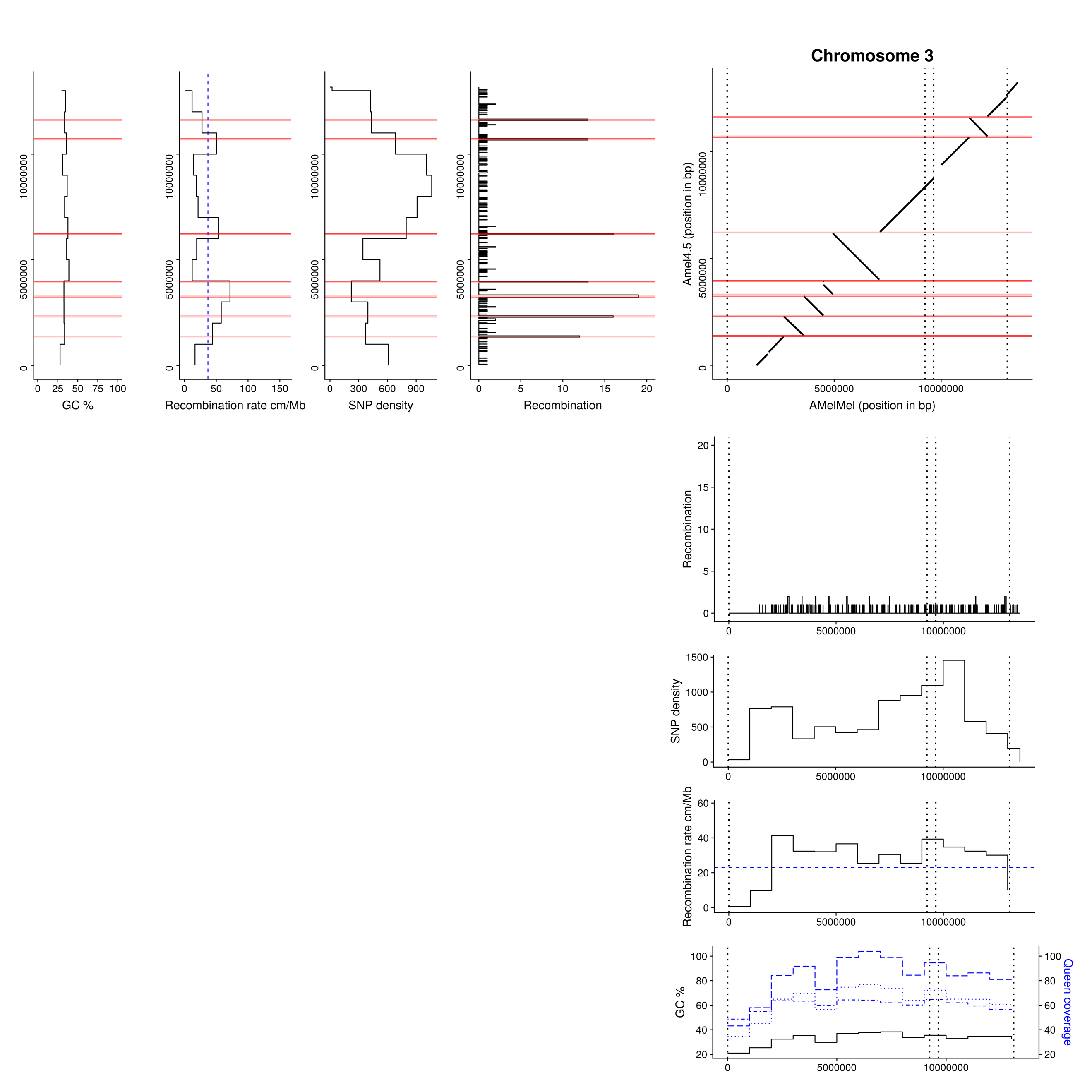


**Subpanel 3:** Chromosome 3.


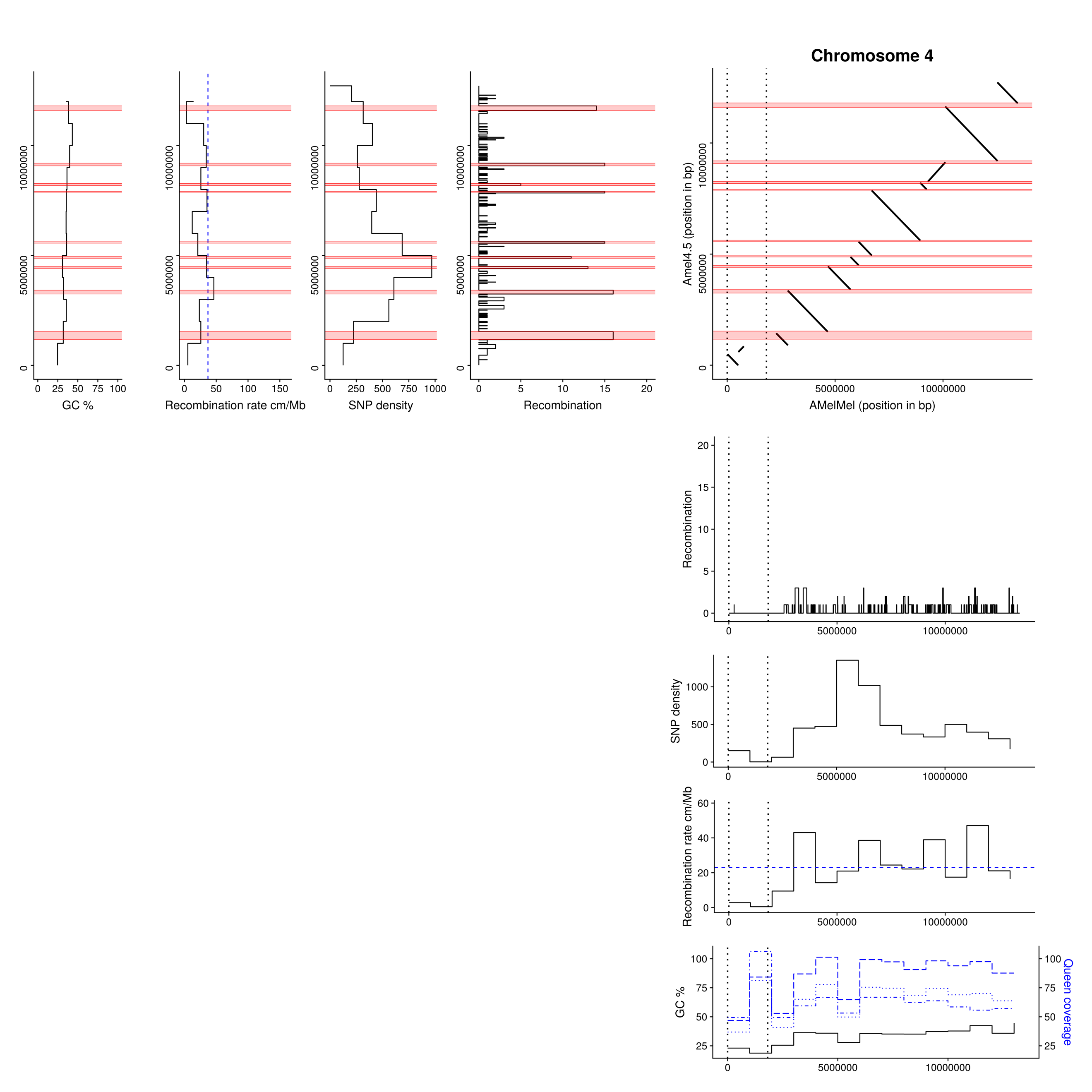


**Subpanel 4:** Chromosome 4.


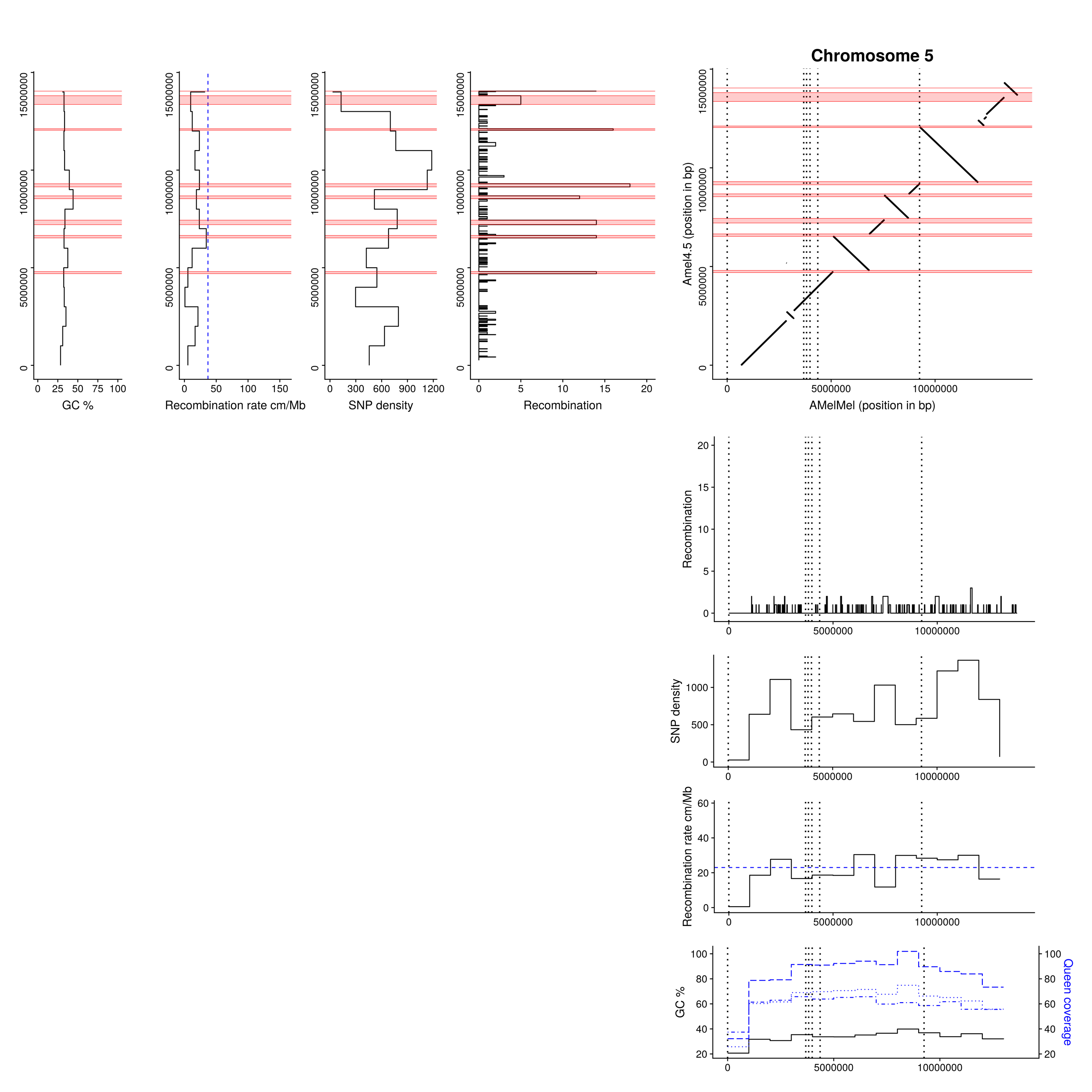


**Subpanel 5:** Chromosome 5.


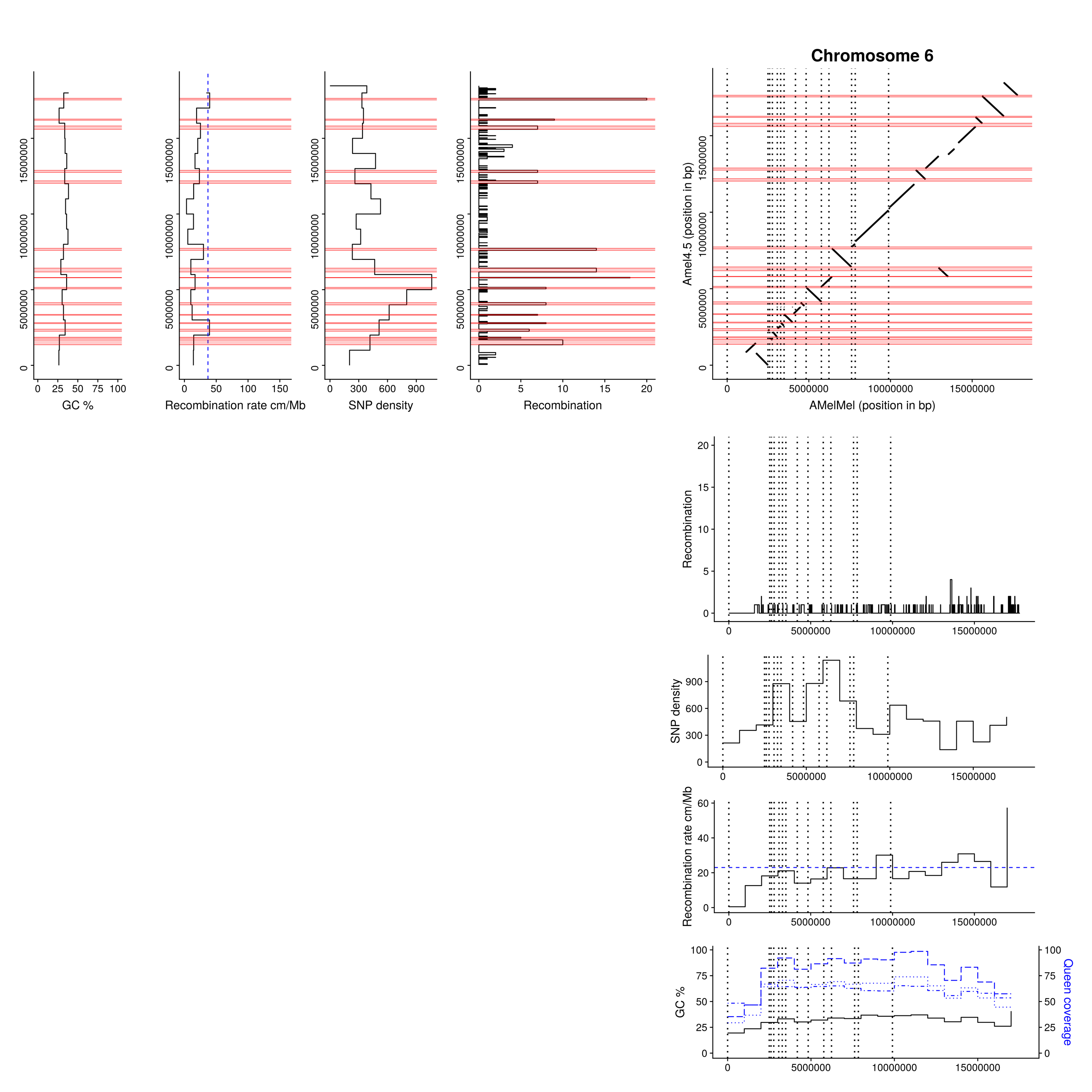


**Subpanel 6:** Chromosome 6.


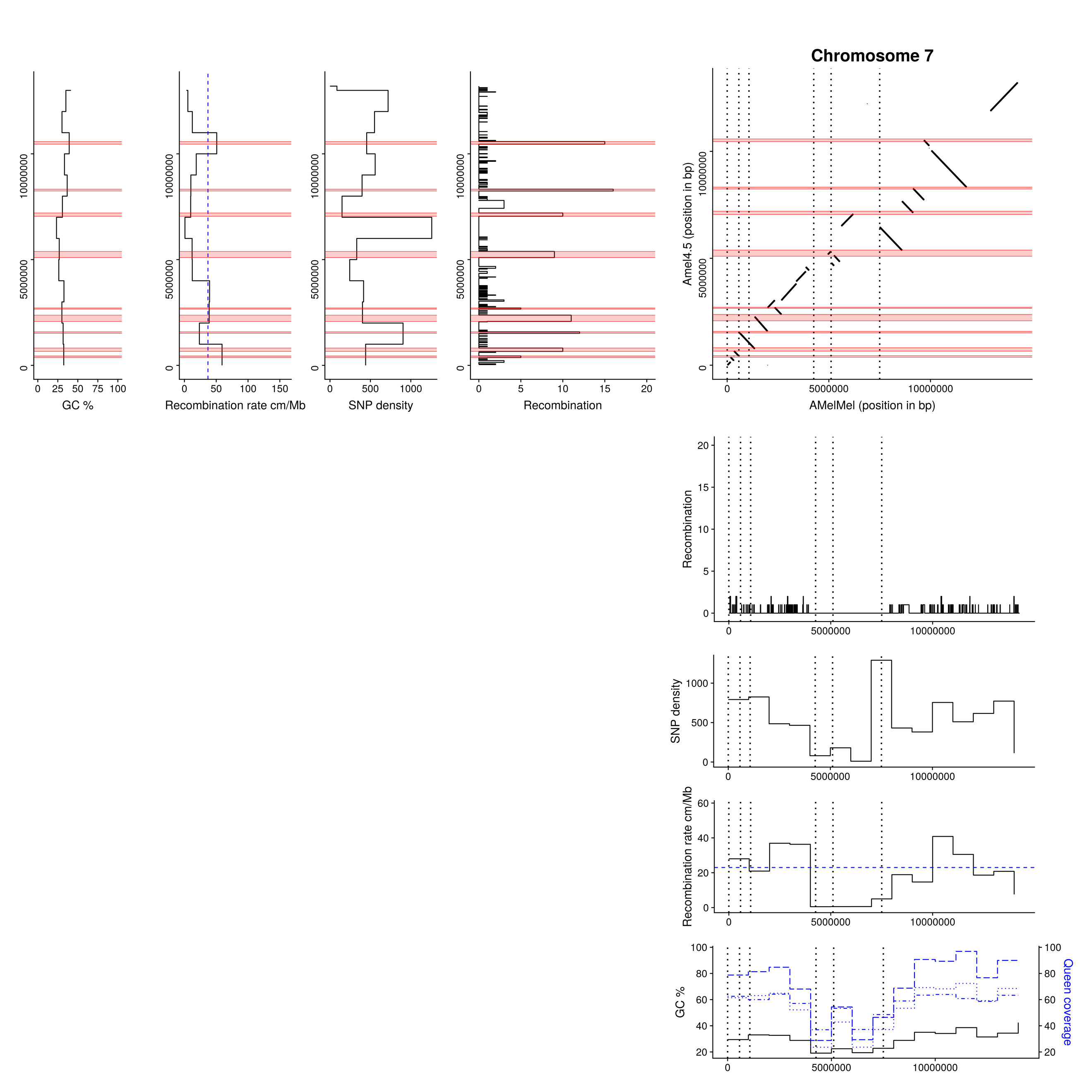


**Subpanel 7:** Chromosome 7.


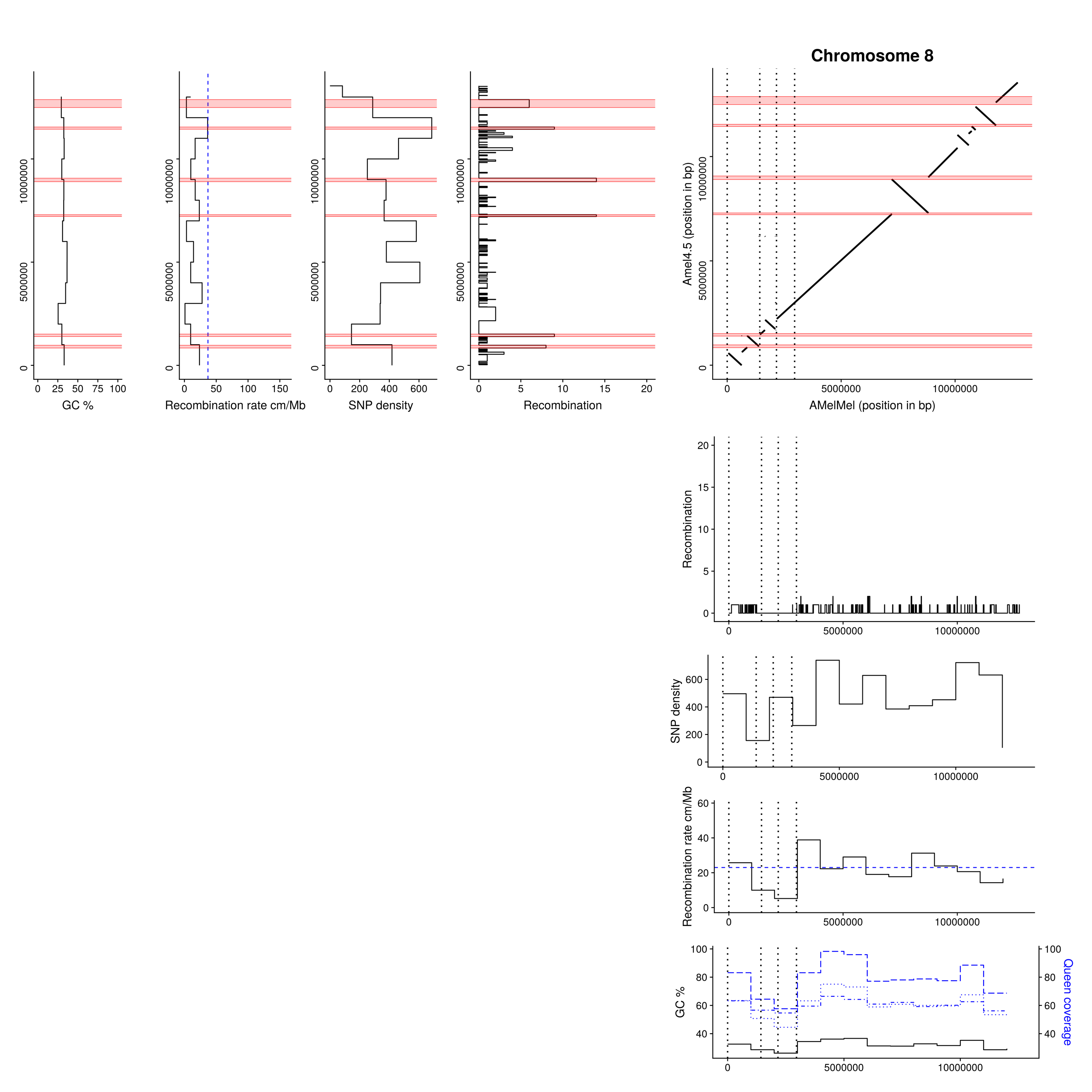


**Subpanel 8:** Chromosome 8.


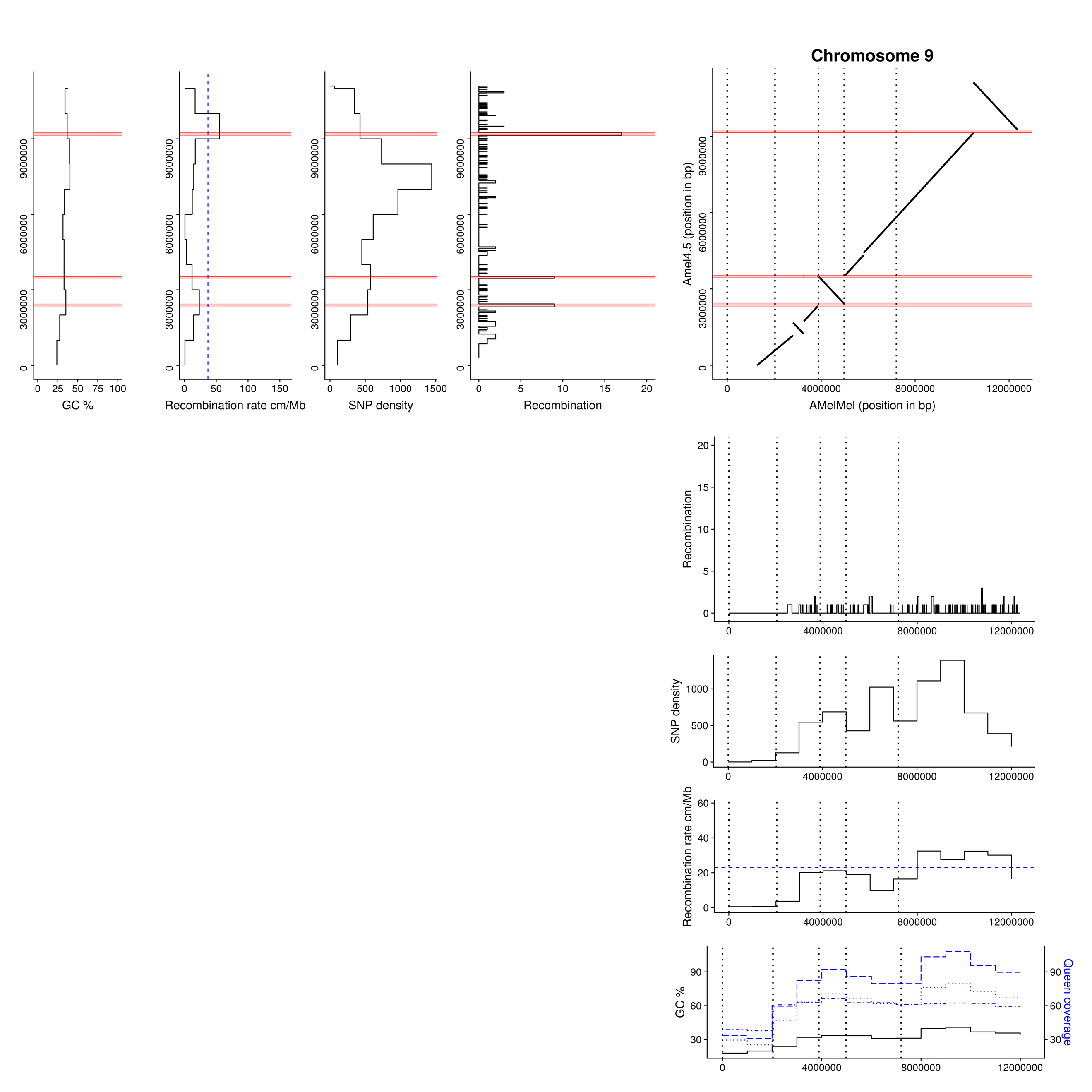


**Subpanel 9:** Chromosome 9.


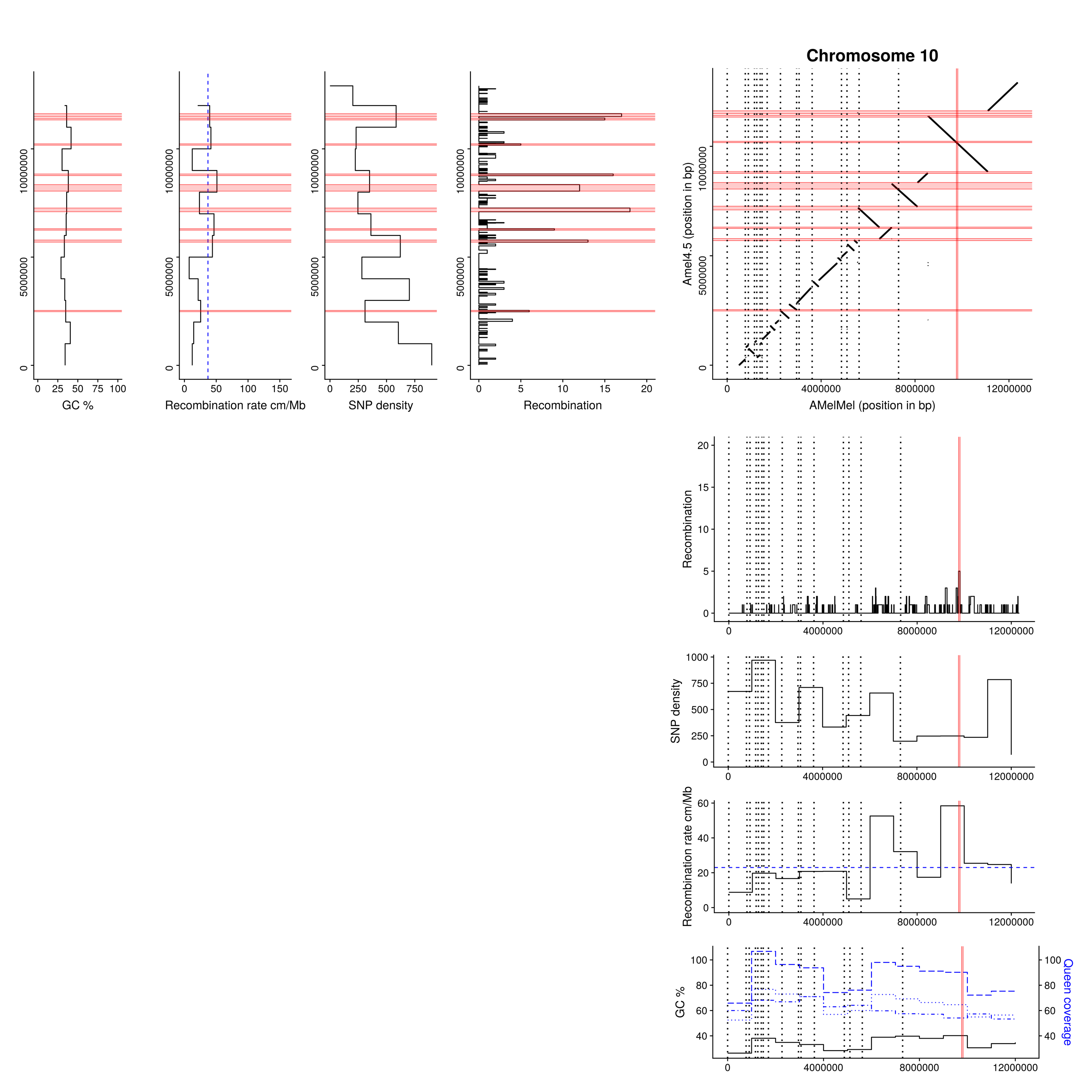


**Subpanel 10:** Chromosome 10.


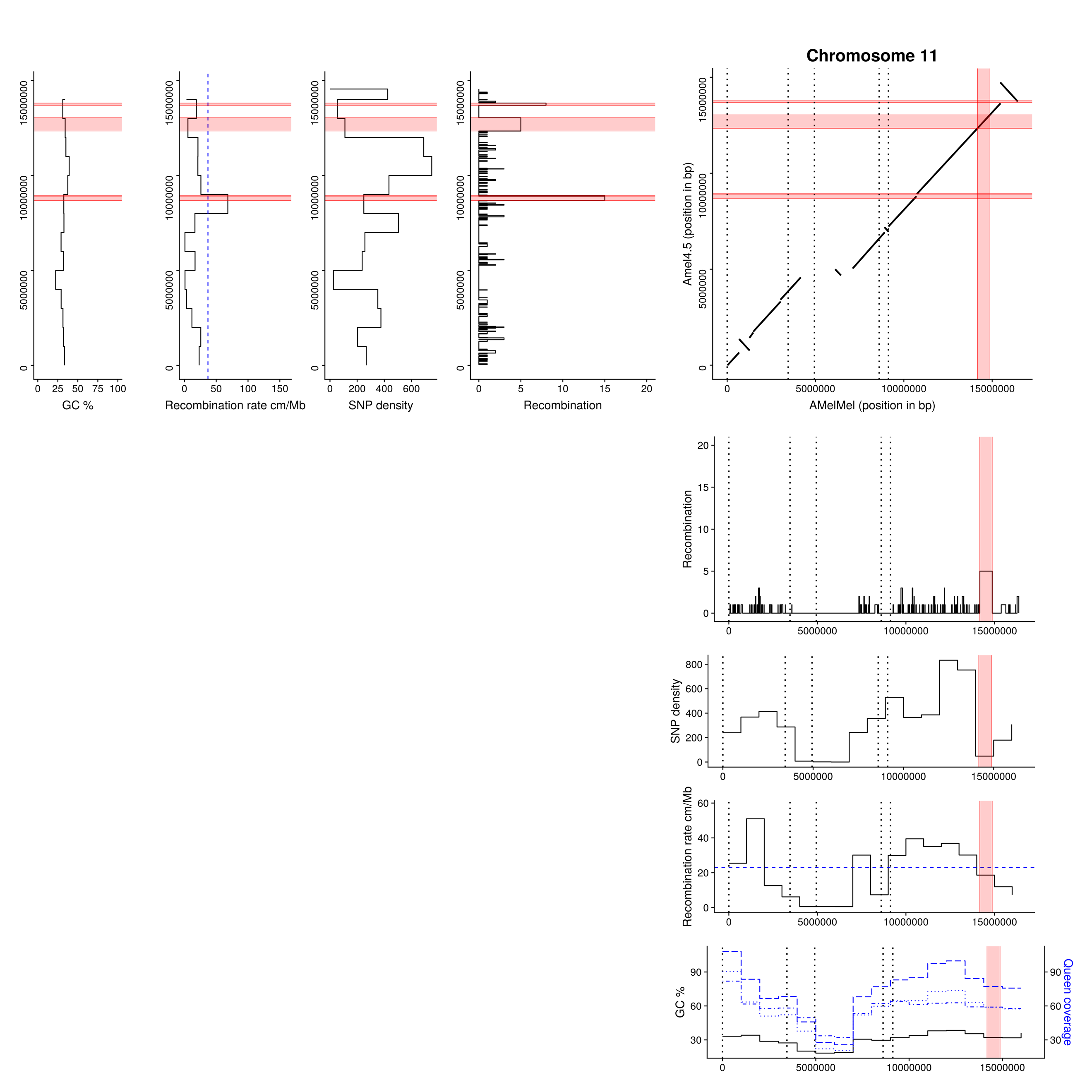


**Subpanel 11:** Chromosome 11.


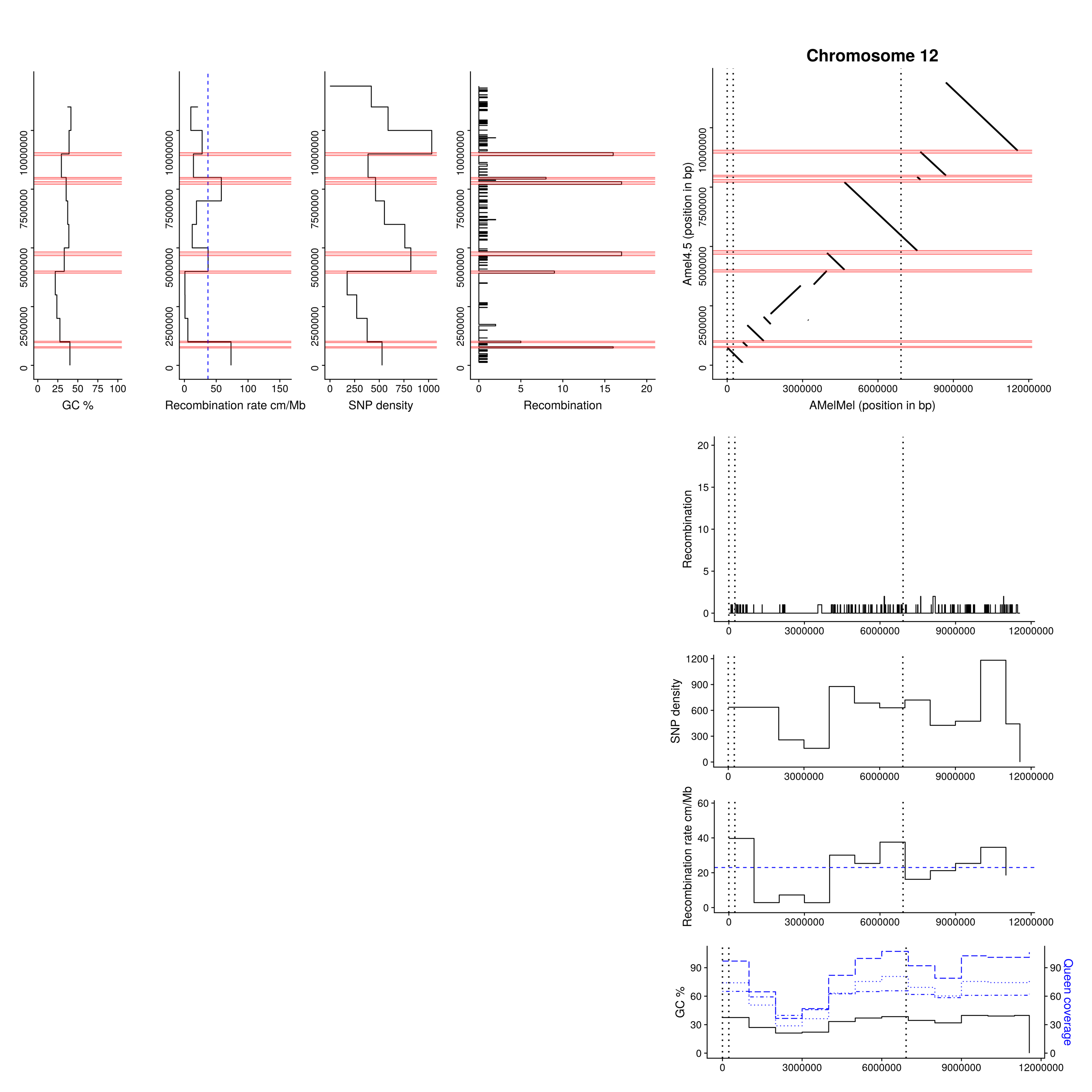


**Subpanel 12:** Chromosome 12.


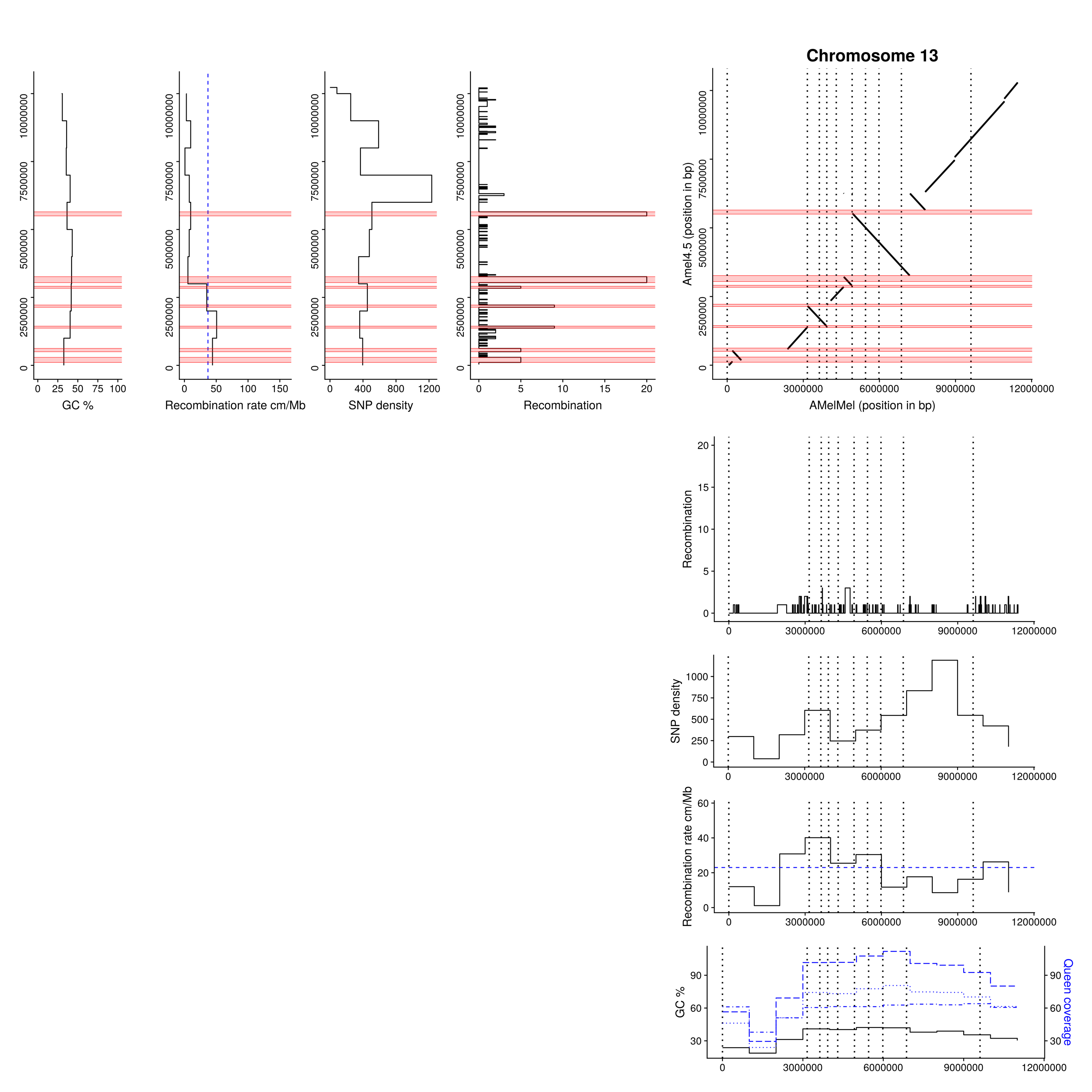


**Subpanel 13:** Chromosome 13.


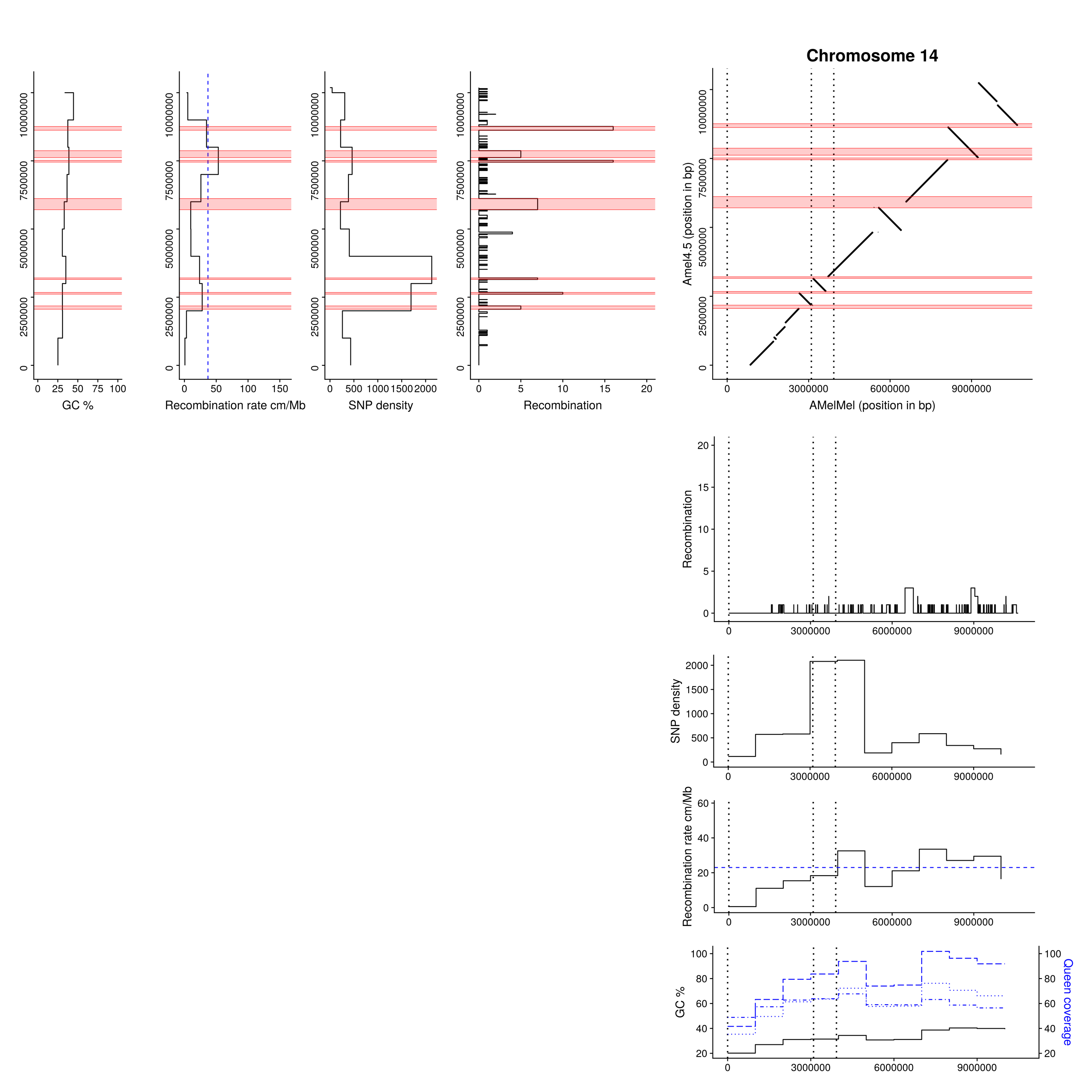


**Subpanel 14:** Chromosome 14.


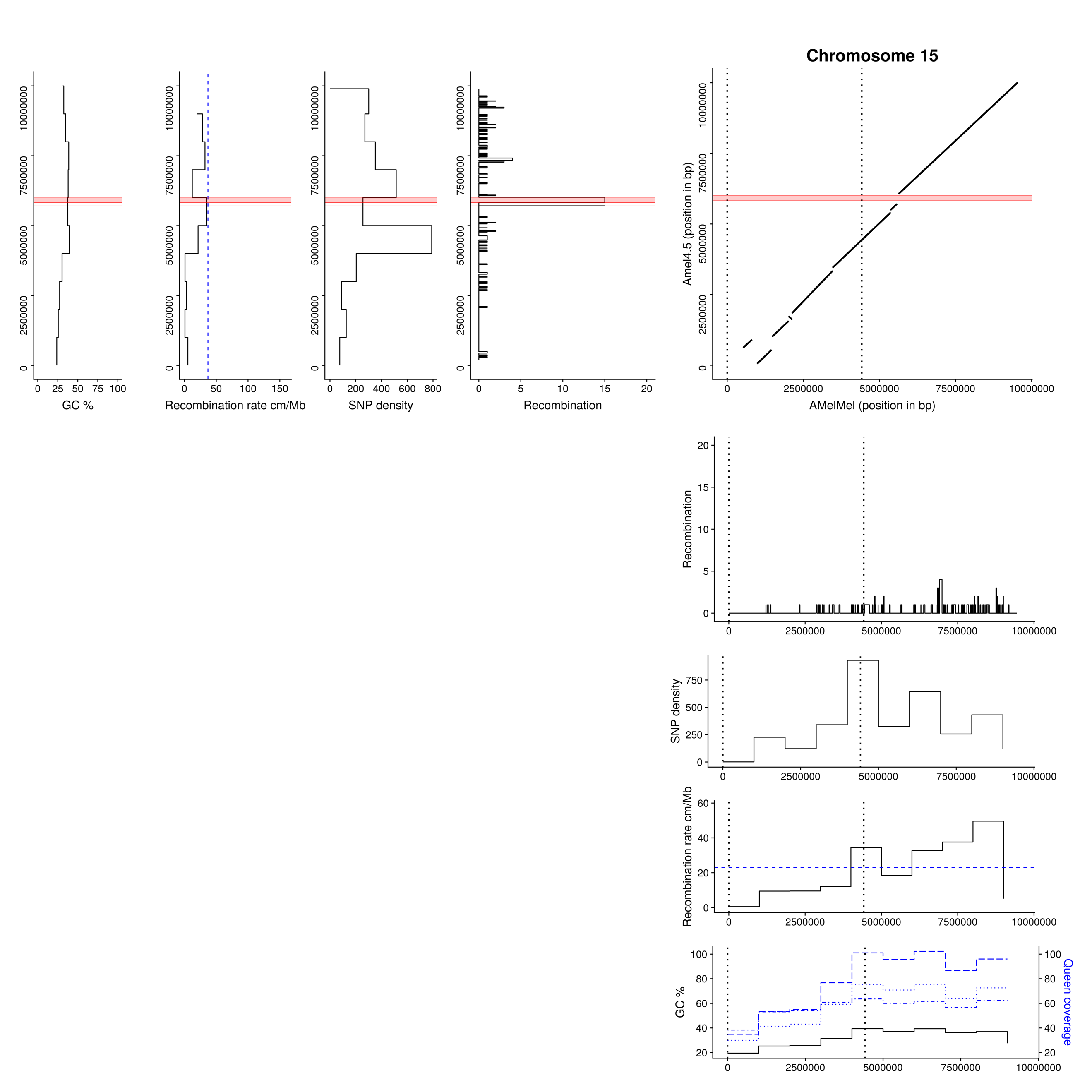


**Subpanel 15:** Chromosome 15.


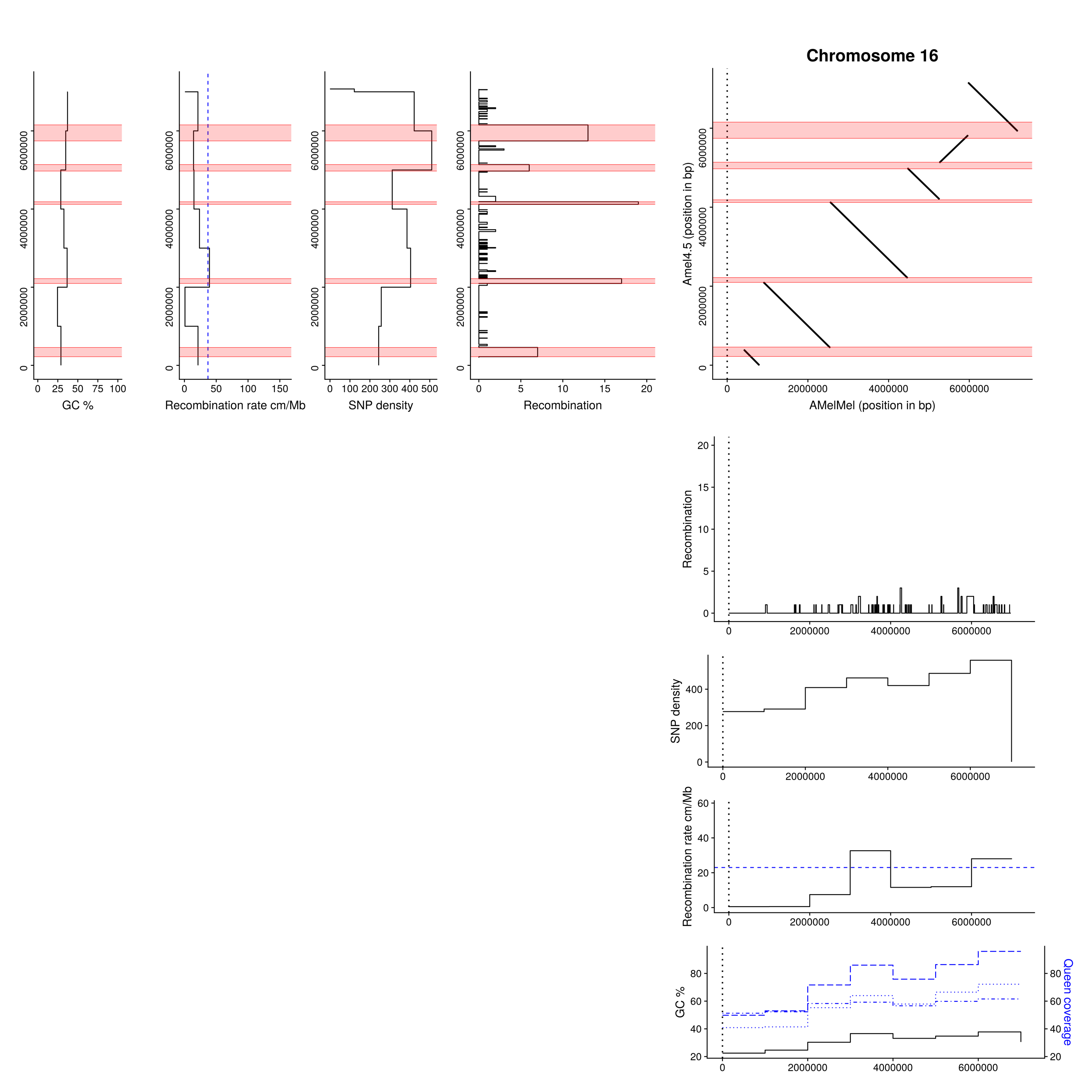


**Subpanel 16:** Chromosome 16.
