# Supplementary material for: The black honey bee genome: insights on specific structural elements and a first step towards pangenomes
Source: Genet Sel Evol. 2024 Jun 28;56:51. doi: 10.1186/s12711-024-00917-3 (PMC11212449; doi:10.1186/s12711-024-00917-3)
Supplement: Supplementary file 5 — Additional file 5. Figure S24. Inversions larger than 1 kb detected between the AMelMel1.1 and HAv3.1 genome assemblies. [file 12711_2024_917_MOESM5_ESM.docx]

Additional file 5:

**Figure S24: Inversions larger than 1 kb between AmelMel1.1 and Hav3.1 genome assemblies.** Inversion events were detected by LAST and distant from contig breakpoints


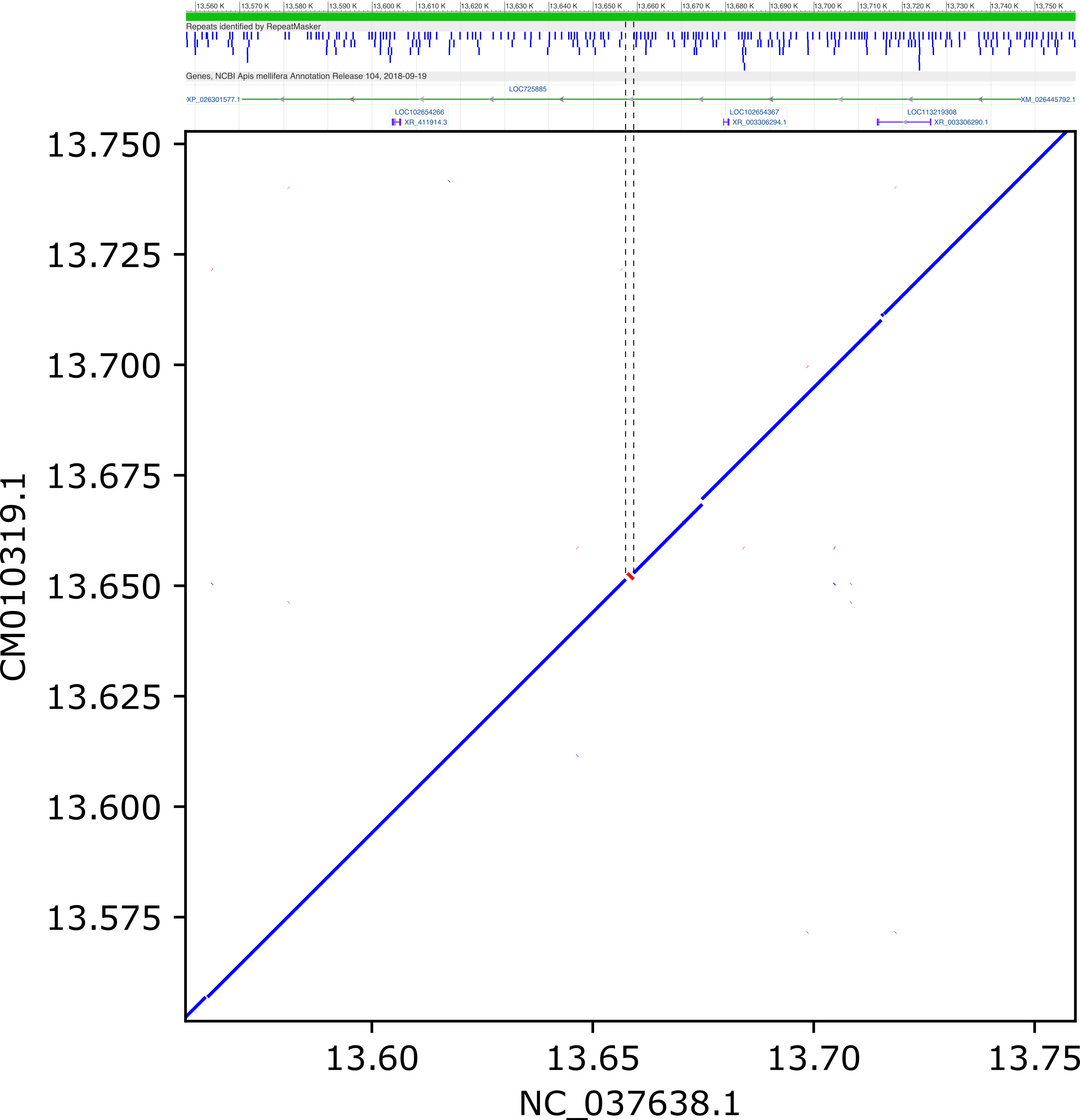


**Subpanel 1:** Chromosome 1


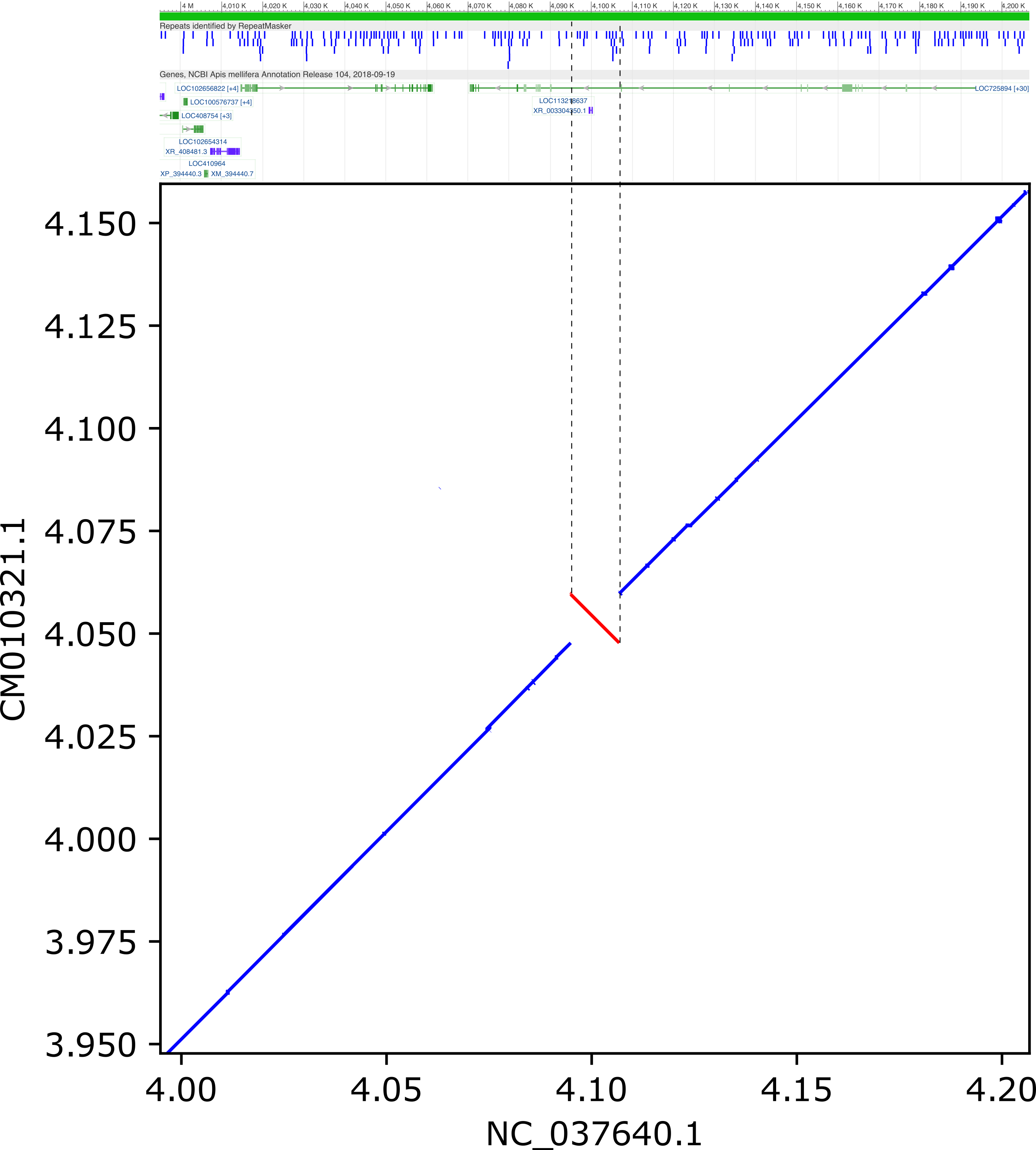


**Subpanel 2:** Chromosome 3**,**


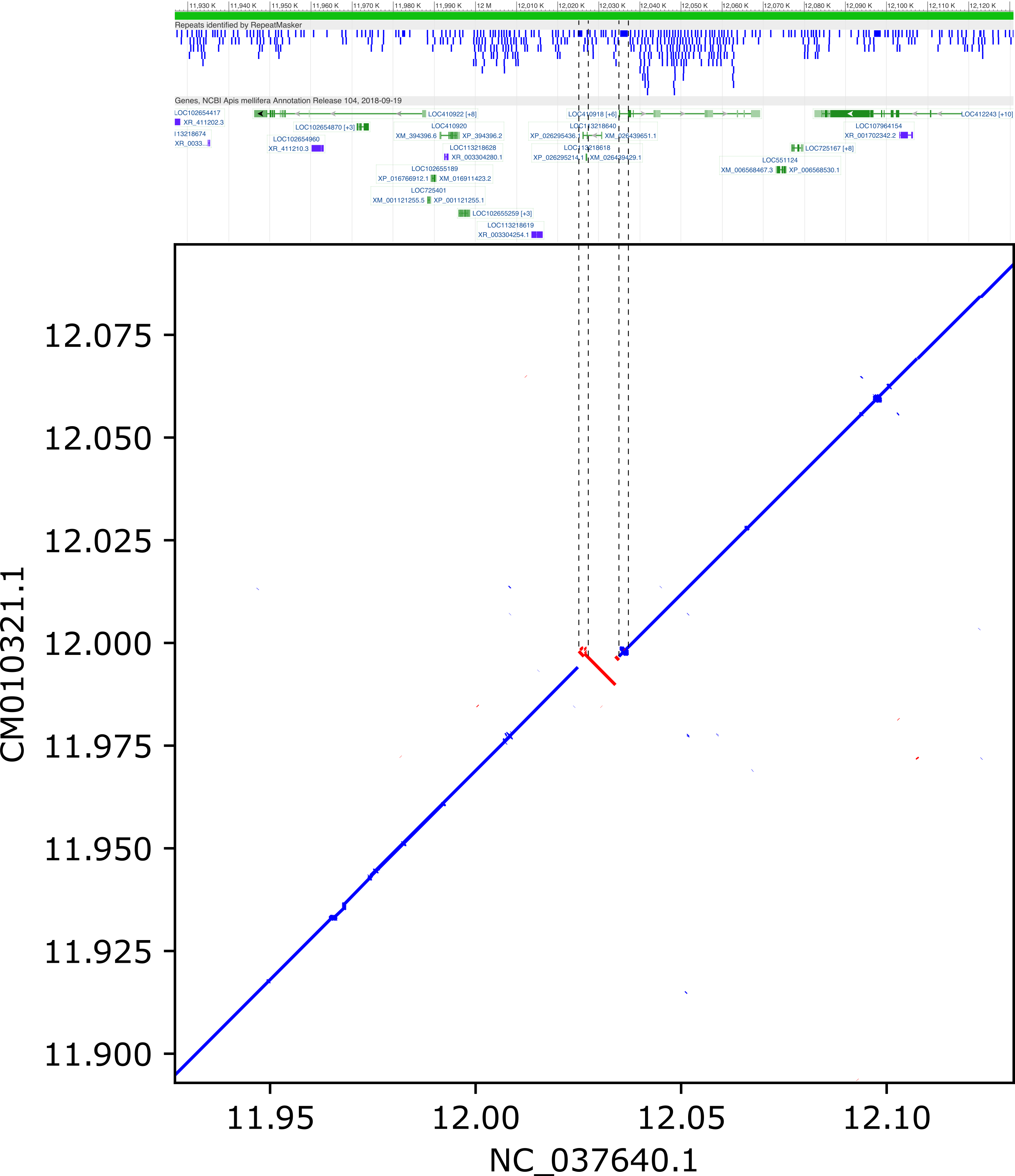


**Subpanel 3:** Chromosome 3


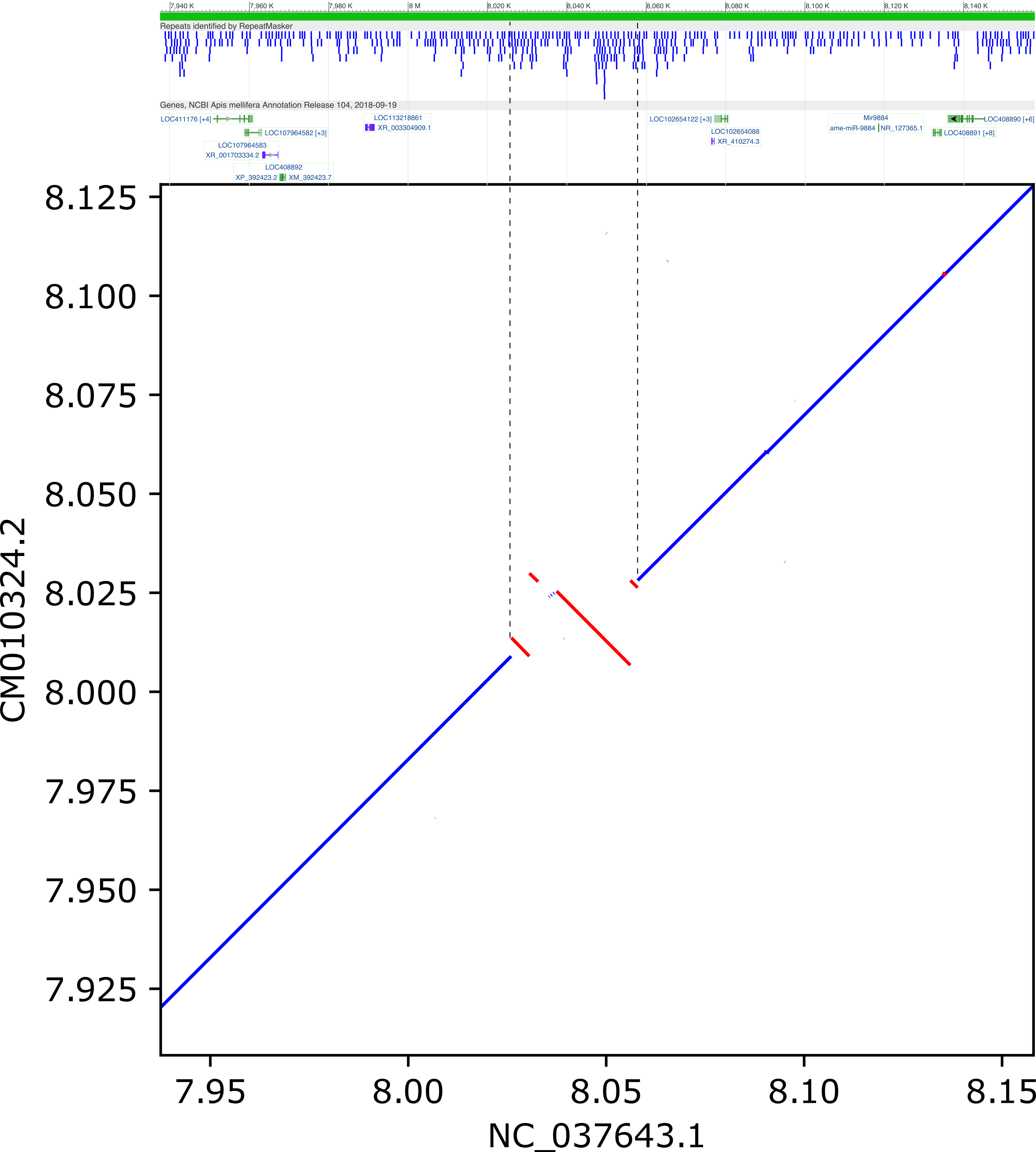


**Subpanel 4:** Chromosome 6


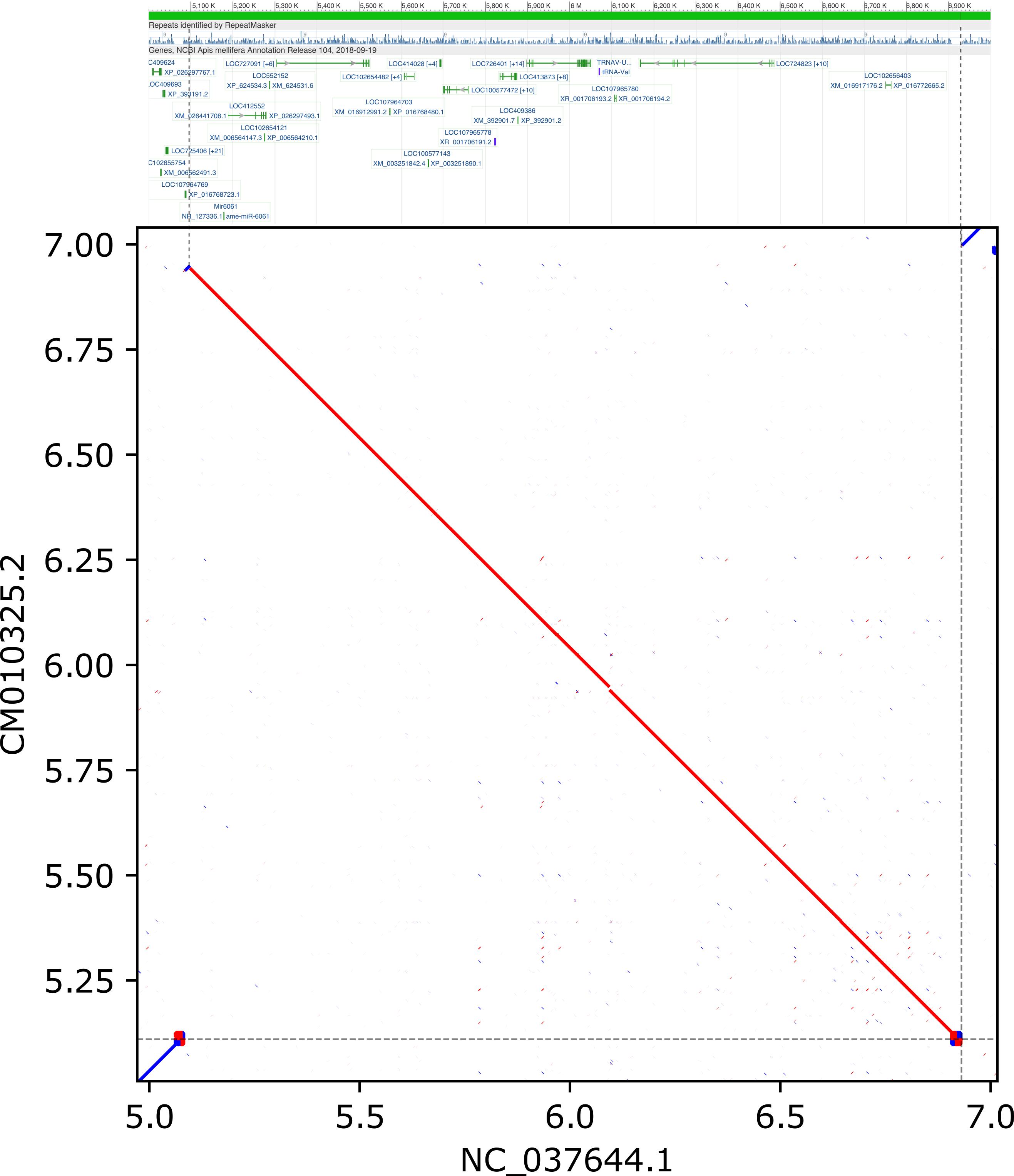


**Subpanel 5:** Chromosome 7


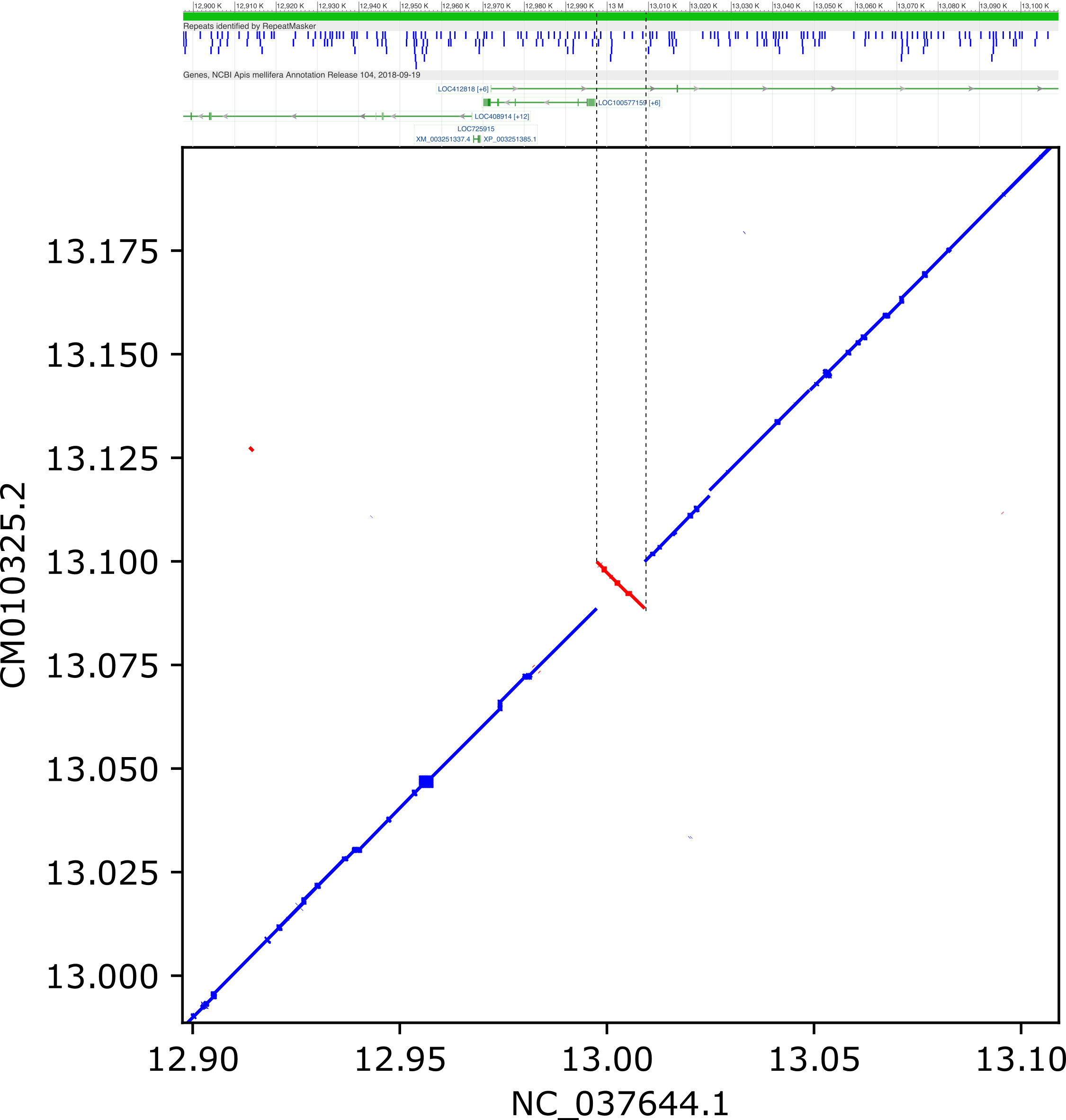


**Subpanel 6:** Chromosome 7


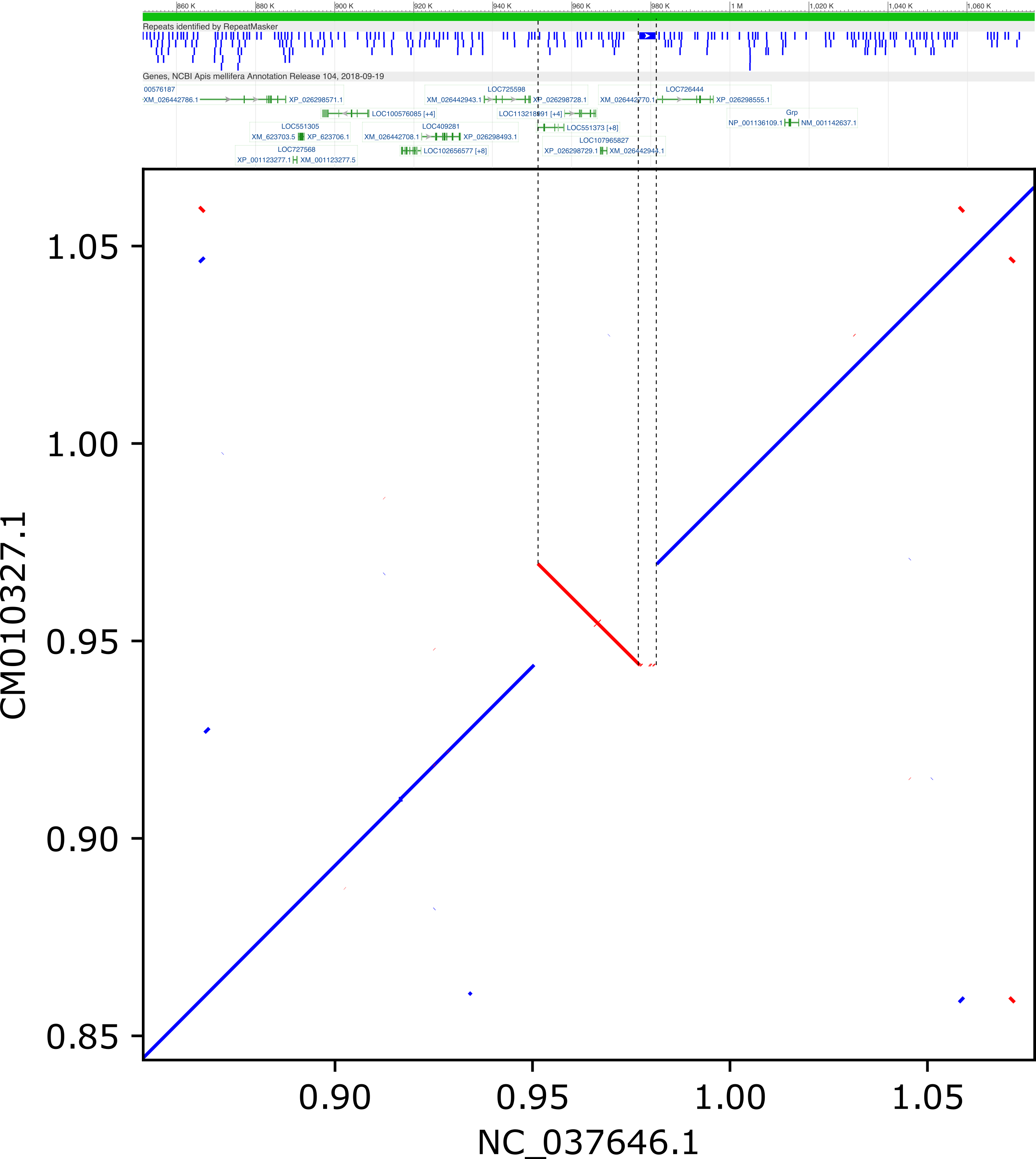


**Subpanel 7:** Chromosome 9


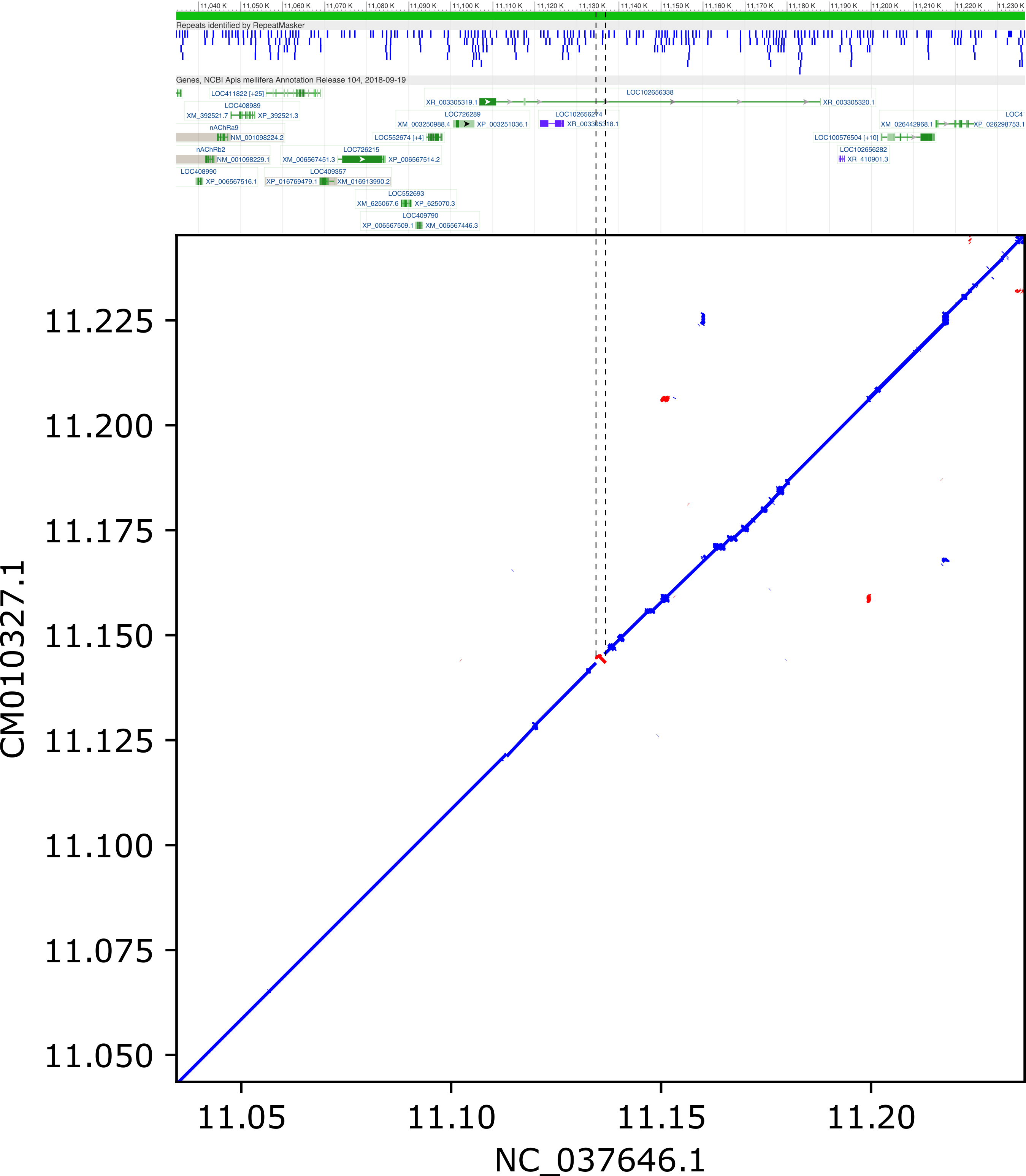


**Subpanel 8:** Chromosome 9


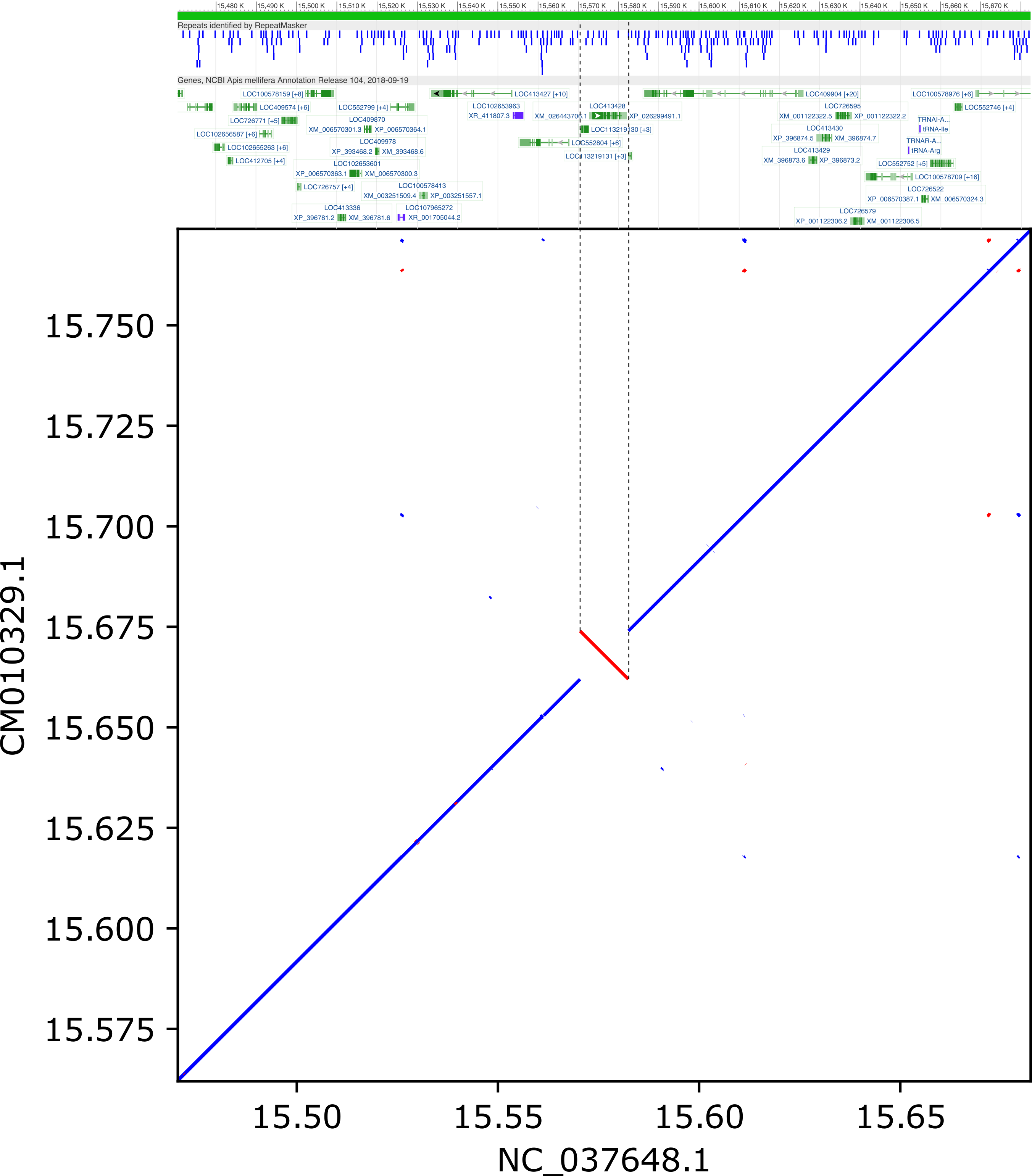


**Subpanel 9:** Chromosome 11


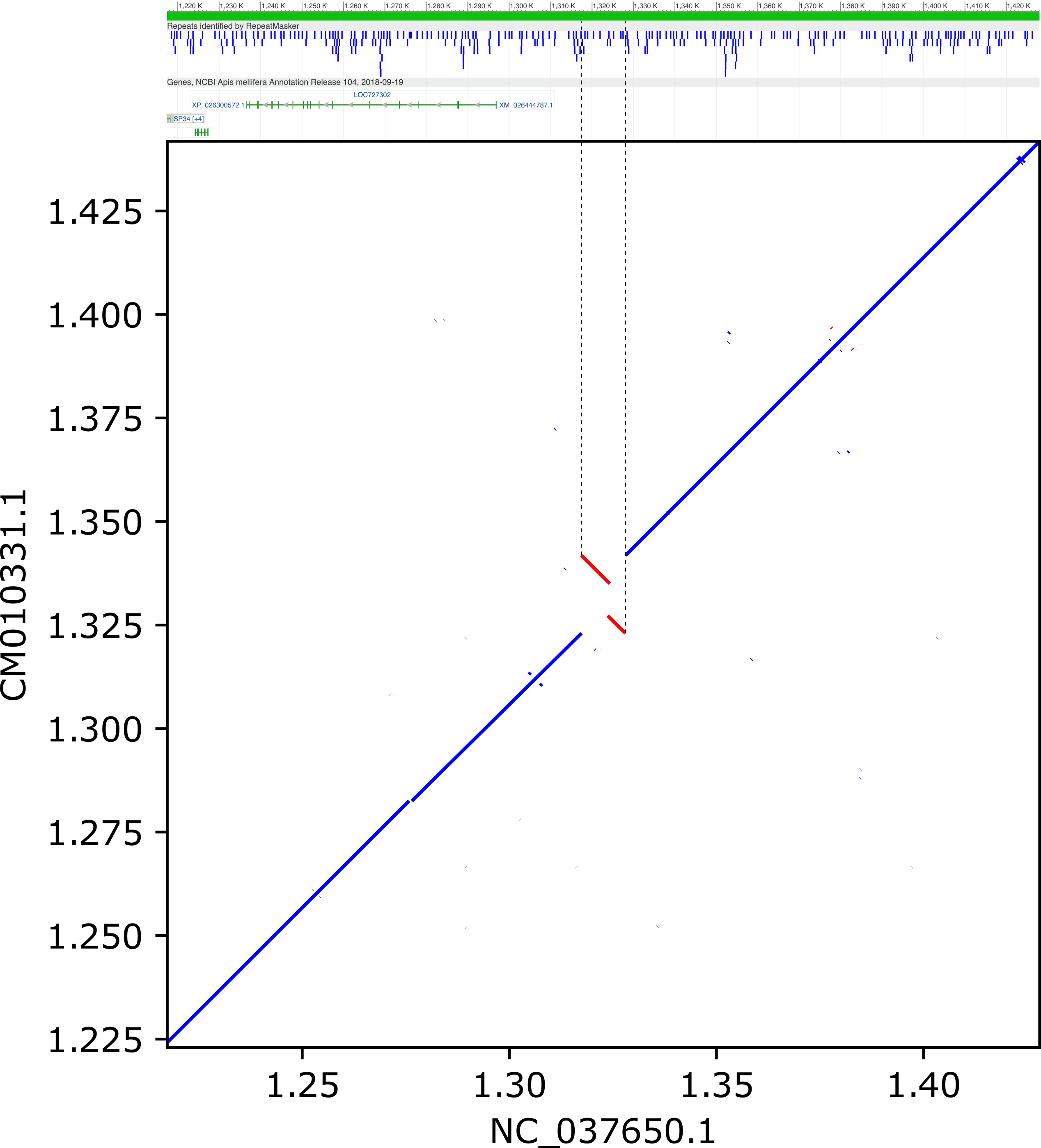


**Subpanel 10:** Chromosome 13


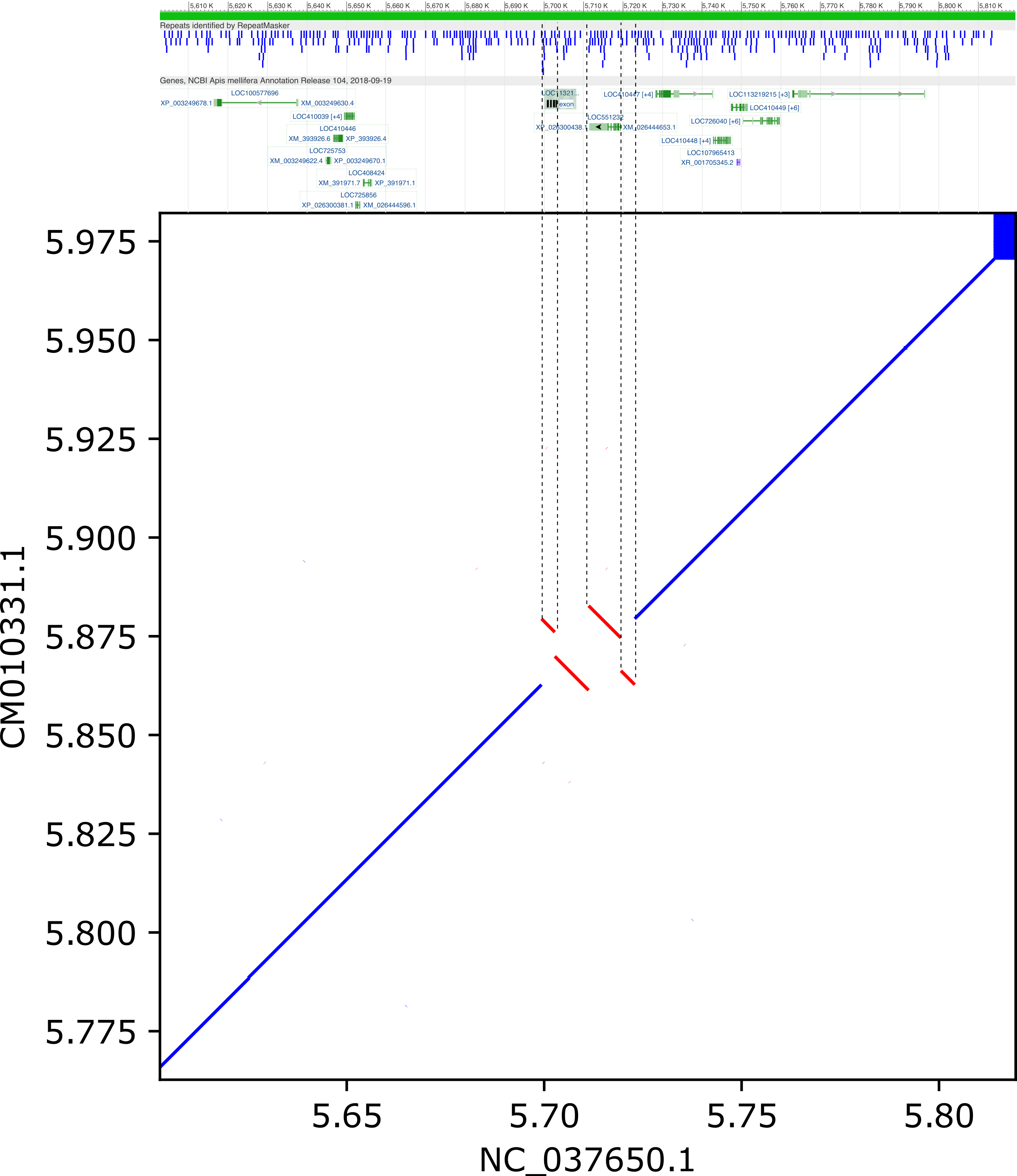


**Subpanel 11:** Chromosome 13


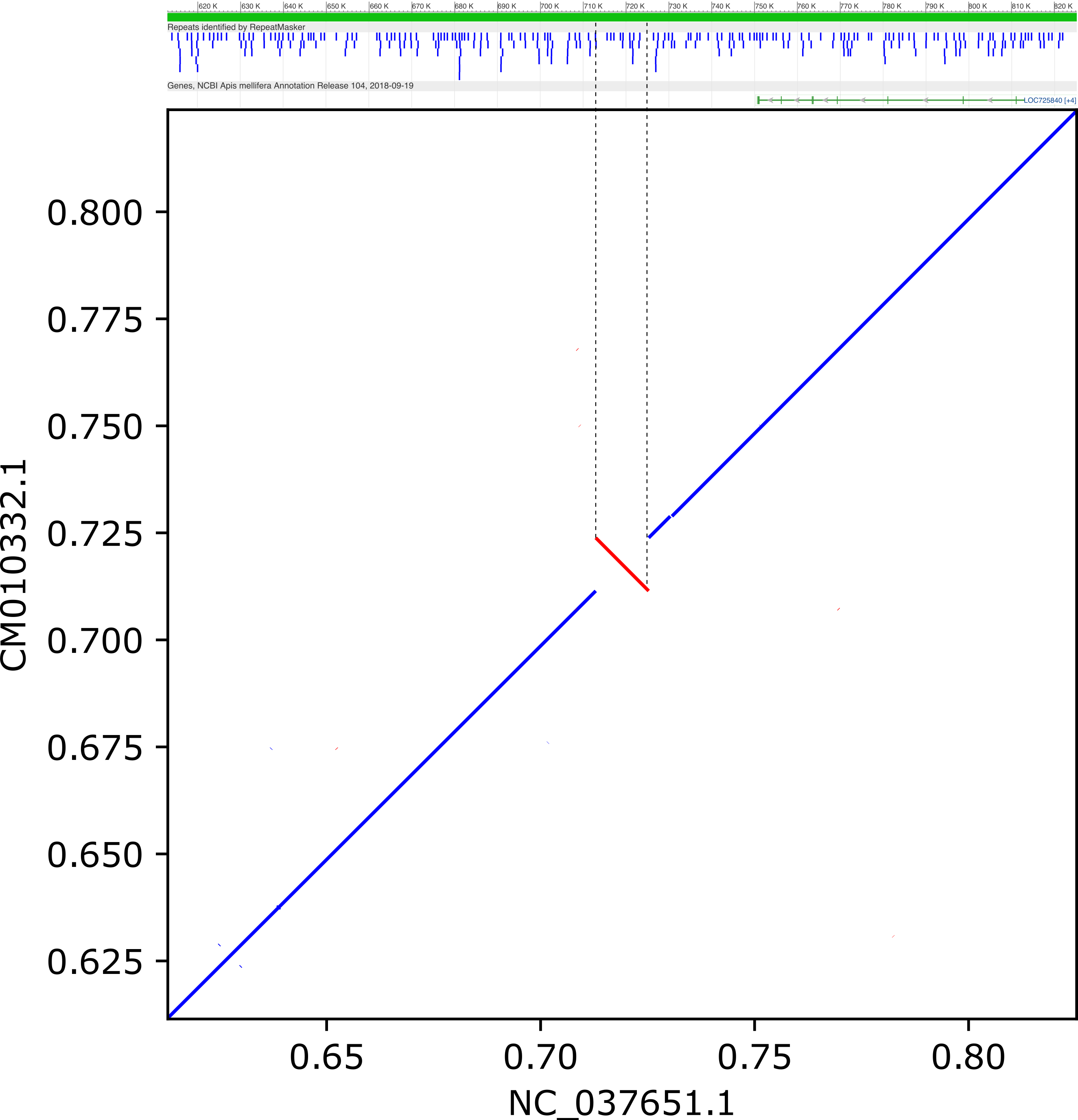


**Subpanel 12:** Chromosome 14


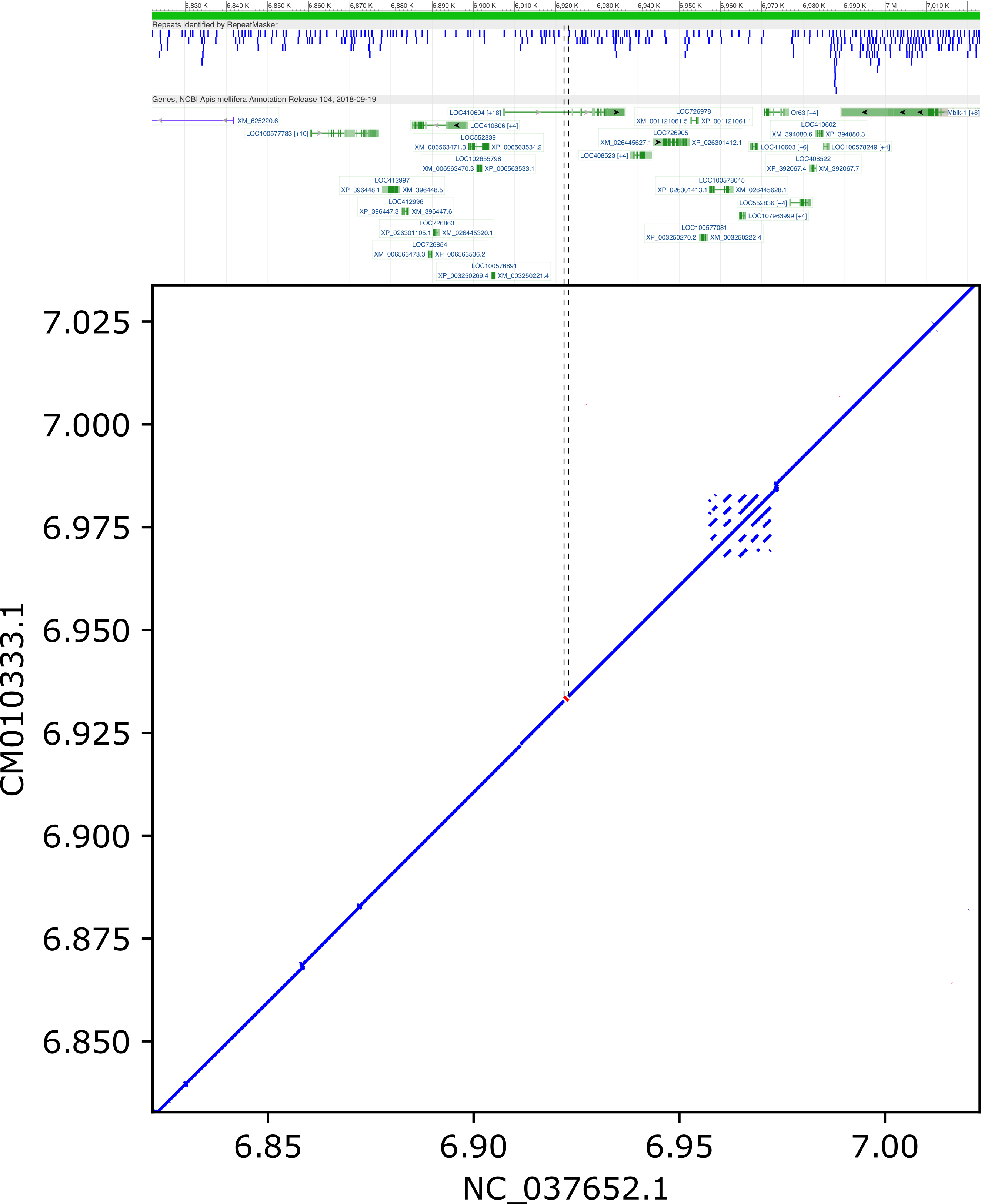


**Subpanel 13:** Chromosome 15
